# DC vaccines loaded with glioma cells killed by photodynamic therapy induce Th17 anti-tumor immunity and provide a four-gene signature for glioma prognosis

**DOI:** 10.1038/s41419-022-05514-0

**Published:** 2022-12-21

**Authors:** Maria Vedunova, Victoria Turubanova, Olga Vershinina, Maria Savyuk, Iuliia Efimova, Tatiana Mishchenko, Robrecht Raedt, Anne Vral, Christian Vanhove, Daria Korsakova, Claus Bachert, Frauke Coppieters, Patrizia Agostinis, Abhishek D. Garg, Mikhail Ivanchenko, Olga Krysko, Dmitri V. Krysko

**Affiliations:** 1 Institute of Biology and Biomedicine, National Research Lobachevsky State University of Nizhny Novgorod, Nizhny Novgorod, Russia; 2 Cell Death Investigation and Therapy (CDIT) Laboratory, Department of Human Structure and Repair, Ghent University, Ghent, Belgium; 3 Institute of Information Technology, Mathematics and Mechanics, National Research Lobachevsky State University of Nizhny Novgorod, Nizhny Novgorod, Russia; 4 Cancer Research Institute Ghent, Ghent, Belgium; 5 4Brain Team, Department of Head and Skin, Ghent University, Ghent, Belgium; 6 Radiobiology Research Group, Department of Human Structure and Repair, Ghent University, Ghent, Belgium; 7 IBiTech-MEDISIP-Infinity Laboratory, Department of Electronics and Information Systems, Ghent University, Ghent, Belgium; 8 Upper Airways Research Laboratory, Department of Head and Skin, Ghent University, Ghent, Belgium; 9 Center for Medical Genetics Ghent (CMGG), Department of Biomolecular Medicine, Ghent University, Ghent, Belgium; 10 Laboratory of Cell Death Research & Therapy, Department of Cellular and Molecular Medicine, KU Leuven, Leuven, Belgium; 11 VIB Center for Cancer Biology Research, Leuven, Belgium; 12 Laboratory of Cell Stress & Immunity (CSI), Department of Cellular & Molecular Medicine, KU Leuven, Leuven, Belgium

**Keywords:** CNS cancer, Preclinical research, Cancer immunotherapy, Cell death and immune response, Prognostic markers

## Abstract

Gliomas, the most frequent type of primary tumor of the central nervous system in adults, results in significant morbidity and mortality. Despite the development of novel, complex, multidisciplinary, and targeted therapies, glioma therapy has not progressed much over the last decades. Therefore, there is an urgent need to develop novel patient-adjusted immunotherapies that actively stimulate antitumor T cells, generate long-term memory, and result in significant clinical benefits. This work aimed to investigate the efficacy and molecular mechanism of dendritic cell (DC) vaccines loaded with glioma cells undergoing immunogenic cell death (ICD) induced by photosens-based photodynamic therapy (PS-PDT) and to identify reliable prognostic gene signatures for predicting the overall survival of patients. Analysis of the transcriptional program of the ICD-based DC vaccine led to the identification of robust induction of Th17 signature when used as a vaccine. These DCs demonstrate retinoic acid receptor-related orphan receptor-γt dependent efficacy in an orthotopic mouse model. Moreover, comparative analysis of the transcriptome program of the ICD-based DC vaccine with transcriptome data from the TCGA-LGG dataset identified a four-gene signature (CFH, GALNT3, SMC4, VAV3) associated with overall survival of glioma patients. This model was validated on overall survival of CGGA-LGG, TCGA-GBM, and CGGA-GBM datasets to determine whether it has a similar prognostic value. To that end, the sensitivity and specificity of the prognostic model for predicting overall survival were evaluated by calculating the area under the curve of the time-dependent receiver operating characteristic curve. The values of area under the curve for TCGA-LGG, CGGA-LGG, TCGA-GBM, and CGGA-GBM for predicting five-year survival rates were, respectively, 0.75, 0.73, 0.9, and 0.69. These data open attractive prospects for improving glioma therapy by employing ICD and PS-PDT-based DC vaccines to induce Th17 immunity and to use this prognostic model to predict the overall survival of glioma patients.

## Introduction

Gliomas, the most frequent intrinsic type of primary tumors of the central nervous system (CNS) in adults, are associated with significant morbidity and mortality [1]. According to the newest World Health Organization classification of tumors of the CNS [2], gliomas, glioneuronal tumors, and neuronal tumors are divided into six different families. Among these are adult-type diffuse gliomas (i.e., most adult patients with primary brain tumors, e.g., glioblastoma (GBM), IDH- wildtype), pediatric-type diffuse low-grade gliomas (with favorable prognoses), and pediatric-type diffuse high-grade gliomas (with poor prognoses) [3]. The pediatric and adult types of gliomas are distinctively different biologically and genetically. Of note, pediatric-type diffuse gliomas have been subdivided into low-grade gliomas (LGG) and high-grade gliomas (HGG) [4]. GBM is classified as a grade 4 malignancy; it is the most aggressive type of cancer of the central nervous system and has a poorer prognosis [3, 5, 6]. Despite the development of novel, complex, multidisciplinary, targeted therapies, such as focal radiotherapy and adjuvant chemotherapeutics in combination with surgical resection, glioblastoma therapy has not progressed much over the last decades [7]. The median survival of patients diagnosed with glioblastoma is 12–15 months, with a five-year survival rate of 5% [8, 9]. Therefore, there is an urgent need to develop novel patient-adjusted anticancer immunotherapies that actively stimulate antitumor T cells, generate long-term memory, and result in significant clinical benefits.

Several recent, novel, therapeutic approaches have emerged that rely on vaccination to activate the patient’s own immune system and to induce a potent and long-lasting immune response against cancer antigens. Dendritic cells (DCs) are key to initiating and directing immune responses [10, 11], and one of these approaches involves the use of DCs loaded with antigenic material derived from or based on the autologous tumor. One such approach is based on the identification of neo-antigens, but it has low efficacy due to the high antigenic heterogeneity of glioma (e.g., glioblastoma multiforme) [12]. Moreover, this approach is complex, labor-intensive, and costly. In contrast, the preparation of cancer cell lysate from the glioma tissue of a patient is less complex and the lysate includes neo-antigens as well as non-mutated tumor antigens, which can result in a broader immune response. However, though the immunogenicity of the lysate loaded in the DCs is important [13–15], whole glioma cells are usually killed by freeze-thawing (F/T) [16, 17], which induces an accidental and unregulated form of necrotic cell death of low immunogenicity [18–20]. One way to increase the immunogenicity of the lysate is to kill the glioma cells by a method that induces immunogenic cell death (ICD) [21, 22].

ICD has recently been shown to be a prerequisite for the activation of the patient’s immune system. Thus, induction of ICD provides two benefits, effectively killing cancer cells and activating an immune response specific for the cancer cells. ICD is characterized by the release or surface exposure of damage-associated molecular patterns (DAMPs), which function as adjuvants to activate strong anticancer immunity [23–25]. Lately, photodynamic therapy (PDT) has been added to the list of therapeutic strategies that can induce typical features of ICD [14, 26, 27]. PDT is a two-stage procedure. The cancer cells are first loaded with a specific drug (photosensitizer, PS), which is then activated by light of a specific wavelength corresponding to the absorption spectrum of the photosensitizer. This results in the generation of singlet oxygen (^1^O_2_) and other toxic reactive oxygen species, which are components of ICD-inducing signaling in cancer cells but are not the only components [26, 28, 29]. We recently demonstrated that clinically approved photosensitizers (photosens [PS] and phthalocyanines complexed with aluminum) could be used to efficiently trigger ICD in several cancer cell types, including glioma cells [20]. Nevertheless, though several methods have been developed to induce ICD in glioma, an effective treatment strategy has not been developed yet.

Here, with the aim of increasing the efficacy of glioma therapy, we used several subcutaneous and orthotopic mouse models to investigate the potential of vaccines based on glioma cells undergoing ICD triggered by PS-PDT. RNA-seq analysis showed that glioma cells undergoing ICD after PS-PDT induced a typical Th17 signature in the DC vaccines, which were highly effective in protecting mice against gliomas. Moreover, we show that inhibition of retinoic acid receptor-related orphan receptor-γt (RORγt), a regulator of Th17 responses, significantly decreased the effects of the DC vaccines and shortened mouse survival because it reshaped the tumor microenvironment by depleting IL17 in the tumor. Comparison of the transcriptome program of the DC vaccines loaded with GL261 cells undergoing ICD after PS-PDT with the transcriptome data from The Cancer Genome Atlas (TCGA-LGG) dataset identified a four-gene signature (CFH, GALNT3, SMC4, and VAV3) associated with overall survival of glioma patients. These prognostic four gene signatures for predicting patients’ overall survival were validated on different cohorts of gliomas patients, including the datasets of the Chinese Glioma Genome Atlas (CGGA)-LGG, TCGA-GBM, and CGGA-GBM. Our results demonstrate the novel role of Th17 responses in the protection generated by DC vaccines based on ICD induced by PS-PDT and open promising avenues for the use of the prognostic model to predict the overall survival of glioma patients.

## Results

### Vaccination with glioma GL261 cells undergoing ICD pulsed with PS-PDT is protective in the subcutaneous prophylactic vaccination mouse model

**Fig. 1:**
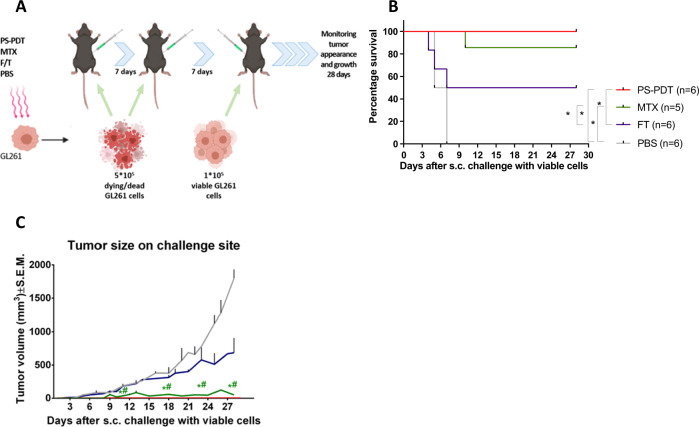
Vaccination with glioma GL261 cells pulsed with PS-PDT in the subcutaneous prophylactic vaccination mouse model. **A** Prophylactic vaccination of mice was performed by injecting them in the left flank on days 0 and 7 with dying/dead GL261 cells treated with three F/T cycles, 2.0 µM MTX, or PS-PDT, or by injecting them with PBS (negative control). Seven days later, the mice were challenged with viable GL261 cells in the right flank. **B** Tumor appearance (% survival) and growth **C** at the challenge site in mice subjected to vaccination and challenge with viable GL261 cells; *n* = 5–6 per group. *Statistically significant difference from the PBS group, (*p* < 0.05); ^#^statistically significant difference from the F/T group, (*p* < 0.05), Wilcoxon test.

We first tested the ability of dying GL261 glioma cells to activate the adaptive immune system by using the gold-standard prophylactic tumor vaccination model in immunocompetent C57BL/6J mice [22]. We adapted the model and subcutaneously vaccinated mice twice with 5 × 10^5^ glioma cells with a one-week interval and then challenged them with 1 × 10^5^ viable glioma cells (Fig. 1A). Tumor growth and appearance were monitored to estimate the success of priming the adaptive immune system. As a positive control, we included mitoxantrone (MTX), a well-known ICD inducer [19, 30] that reduces the risk of death in patients with recurrent GBM [31, 32]. For the negative control (non-ICD), we injected mice either with PBS or with 5 × 10^5^ F/T (accidentally necrotic) mouse glioma GL261 cells. Incubation of glioma GL261 cells with 1.4 µM of the PS and subsequent irradiation with a light dose of 20 J/cm^2^ induced a mixed type of regulated cell death with both apoptotic and ferroptotic features (Suppl. Fig. 1A, B), confirming our previously published findings [20]. The mice immunized twice with glioma GL261 cells treated with PS-PDT (GL261_PS-PDT) showed signs of robust activation of the adaptive immune system, better survival (Fig. 1B), and protection against tumor growth (Fig. 1C) resembling that in mice vaccinated with glioma GL261 cells treated with MTX (GL261_MTX; positive control). Importantly, mice vaccinated with GL261_PS-PDT developed no measurable tumors and all mice survived, indicating that GL261_PS-PDT is strongly immunogenic. Most of the mice immunized with PBS showed extensive tumor growth at the challenge site (Fig. 1B, C). Notably, the mice vaccinated twice with the same number of F/T GL261 cells developed significantly larger tumors (Fig. 1C). These data indicate that glioma GL261 cells undergoing accidental necrosis after F/T have weak immunogenicity and that even priming and boosting the mice with such cells does not provide effective protection against challenge with viable GL261 cells. These data are in agreement with previously published findings using other types of F/T cancer cells for vaccination and indicate that accidentally necrotic cells are less immunogenic [18–20]. Importantly, tumor growth at the challenge site of the unvaccinated (PBS) mice and those vaccinated with F/T glioma GL261 cells (negative control) were significantly larger than the tumors on the mice vaccinated with GL261_PS-PDT (Fig. 1C). However, when the mice were subcutaneously vaccinated only once with 5 × 10^5^ glioma cells and one week later challenged at another site with 1 × 10^5^ viable GL261 glioma cells (Suppl. Fig. 1C), GL261_PS-PDT provided better protection against challenge with viable glioma GL261 cells than in the PBS group (i.e., better survival) though the difference was not statistically significant (Suppl. Fig. 1D). Interestingly, the tumors growing at the challenge site of the unvaccinated (PBS) mice and those vaccinated with F/T glioma GL261 cells (negative control) were significantly larger than the tumors developing on the mice that were vaccinated with GL261_PS-PDT and those in the positive control MTX group, indicating the induction of an immune response in vivo (Suppl. Fig. 1E). Together, these data demonstrate that primer and booster subcutaneous vaccination of mice with GL261_PS-PDT activated anti-tumor immunity.

### Subcutaneous vaccination with glioma GL261 cells pulsed with PS-PDT protects in the orthotopic glioma mouse model

**Fig. 2:**
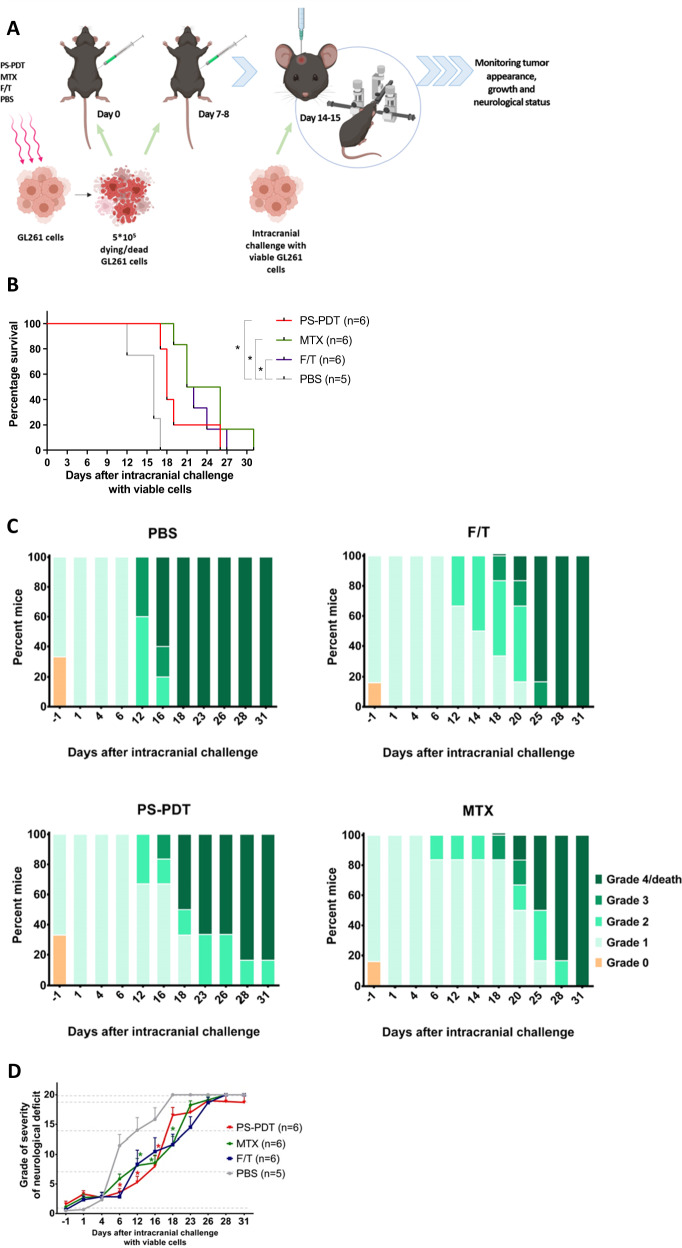
Subcutaneous vaccination with glioma GL261 cells pulsed with PS-PDT protects in the orthotopic high-grade glioma (HGG) mouse model. **A** Experimental setup for the prophylactic vaccination of mice injected with GL261 cells treated with three F/T cycles or 2.0 µM MTX, or loaded with 1.4 µM photosens and exposed to PDT (PS-PDT), or injected with PBS. The mice were vaccinated on days 0 and 7 and challenged by intracranial stereotactic injection of viable GL261 cells on day 14. **B** Survival of mice vaccinated and challenged with GL261 cells as described in (**A**). *p < 0.01, Mantel-Cox logarithmic test. **C**, **D** The percentages of mice showing neurological alterations is shown (grade 0, grade 1, grade 2, grade 3, grade 4). The neurological status of the mice was assessed every 2–4 days for up to day 31 after intracranial tumor inoculation. *N* = 5–6 per group. *p < 0.05, a statistically significant difference from the PBS group; Wilcoxon test.

We examined whether GL261 glioma cells treated with PS-PDT can induce anti-glioma protective immunity in a prophylactic setup in the orthotopic glioma mouse model. Such models are widely used to test different novel experimental treatment strategies and to characterize the immunogenicity of dying glioma cells [14, 27, 33]. Immunocompetent, syngeneic C57BL/6 mice were first subcutaneously vaccinated twice with 5 × 10^5^ glioma cells treated with PS-PDT or MTX, or with GL261 cells subjected to F/T (Fig. 2A). Eight days later, the mice were intracranially (intraventricularly) challenged with 2 × 10^4^ viable glioma GL261 cells and monitored for symptoms of neurological deficit [14, 27, 33] and for survival. Remarkably, the mice immunized twice with GL261_PS-PDT showed signs of activation of anti-tumor immunity and exhibited better survival and protection against intracranial challenge with viable glioma GL261 cells in comparison to mice injected with PBS (Fig. 2B). In the same model, vaccination with GL261_F/T or GL261_MTX also provided considerable protection against challenge with viable GL261 cells, but the results were not significantly different from GL261_PS-PDT vaccination (Fig. 2B). Similarly, analysis of the glioma-induced neurological deficit grades revealed a considerable delay in the onset of clinically relevant symptoms in the mice vaccinated with GL261_PS-PDT as compared to the PBS group (Fig. 2C, D). Of note, a single vaccination with GL261 cells pulsed with GL261_PS-PDT was not protective against intracranial challenge with viable glioma GL261 cells (Suppl. Fig. 2A, B). These data indicate that induction of ICD in glioma GL261 cells by PS-PDT followed by their subcutaneous injection twice induces anti-tumor immunity in the orthotopic glioma mouse model and protects against intra-cranial challenge with viable GL261 cells.

### ICD-based DC vaccines induce significant protective immunity against glioma

**Fig. 3:**
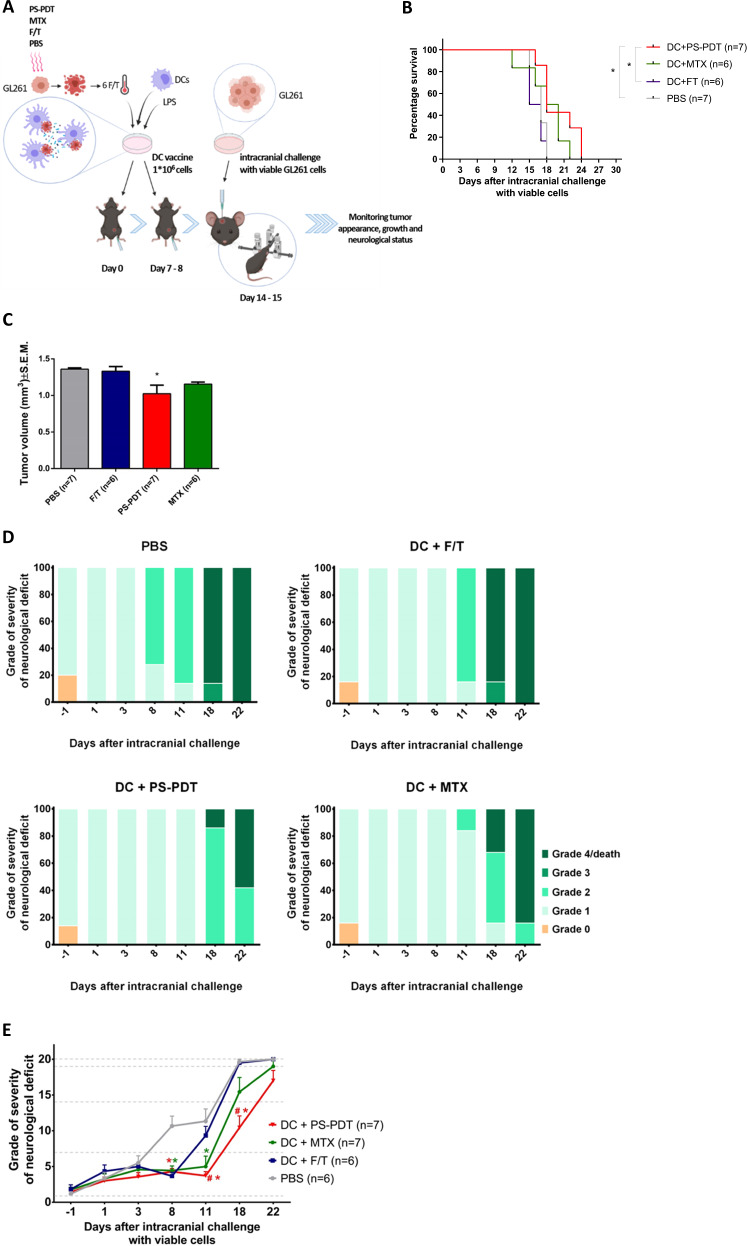
ICD-based DC vaccines provide significant protective immunity against glioma. **A** Experimental setup for the prophylactic vaccination of mice with DC-based vaccines loaded with GL261 cells treated with PS-PDT (PS at a dose of 1.4 µM). As controls, we used DC-based vaccines loaded with GL261 cells subjected to F/T cycles or treated with 2.0 µM MTX or mire were injected with PBS. The mice were injected on days 0 and 7, and seven days after the last vaccination they were intracranially injected with viable GL261 cells using stereotactic coordinates. **B** The curve represents the survival of mice in the four treatment groups of 6–7 mice per group for up to 24 days. Statistical significance was determined by the Mantel-Cox logarithmic test, *p < 0.01. **C** Diffusion-weighted tomography images for determining tumor volume (*n* = 6–7 per group). Statistical significance was determined by unpaired Mann–Whitney *U* test, *p < 0.05. **D**, **E** Temporal progression of neurological deficits in mice treated as described in (**A**). The neurological status of the mice for up to day 22 after intracranial tumor inoculation. The percentage of mice after intracranial tumor inoculation in the DC + PS-PDT or DC + MTX group showed a significant difference in the degree of neurological alterations (grades 0–4); *n* = 6–7 per group; *p < 0.01, a statistically significant difference from the PBS group; ^#^statistically significant difference from the F/T group; Wilcoxon test.

We evaluated the immunogenic potential of GL261_PS-PDT and its ability to trigger anti-glioma protective immunity and examined whether this DC immunotherapy could protect mice against intracranial primary tumor challenge with viable GL261 cells. Indeed, GL261_PS-PDT was efficiently phagocytosed by murine DCs in a ratio-dependent manner (Suppl. Fig. 2C). The rates of engulfment of GL261 glioma cells treated with PS-PDT and those treated with MTX were similar (Suppl. Fig. 2C). These data confirm our previously published findings [20]. Next, immunocompetent syngeneic C57BL/6 mice were vaccinated twice intraperitoneally with DCs loaded ex vivo with GL261_PS-PDT (DC-GL261_PS-PDT) (Fig. 3A). As a positive control, we vaccinated mice with DCs loaded ex vivo with MTX-treated glioma GL261 cells, and for the negative control, we used PBS or DCs loaded ex vivo with F/T glioma GL261 cells. Thereafter, all the mice were intracranially (intraventricularly) inoculated with 2 × 10^4^ live GL261 glioma cells and then monitored the development of symptoms of neurological deficit and survival. Interestingly, the mice vaccinated with DC-GL261_PS-PDT before tumor challenge demonstrated a significant increase in median survival compared to mice injected with PBS (24 days *versus* 18 days, *p* < 0.03) or with DCs loaded with F/T GL261 (24 days *versus* 18 days, *p* < 0.02) (Fig. 3B). Mice vaccinated with DC-GL261_PS-PDT and orthotopically challenged with live GL261 cells showed significantly lower tumor mass than control mice (Fig. 3C). Consistent with overall survival, monitoring of the glioma-induced neurological deficit grades (Fig. 3D, E) revealed not only diminished severity of clinical manifestation but also later onset of symptoms in mice vaccinated with DC-GL261_PS-PDT (18 days versus 8 days). Remarkably, we also observed earlier onset of symptoms in mice vaccinated with DC vaccines loaded with F/T glioma GL261 cells compared to mice vaccinated with DC-GL261_PS-PDT (11 days versus 18 days). All these data indicate that the DC-GL261_PS-PDT vaccine induces anti-tumor immunity in the orthotopic glioma mouse model and protects mice against intra-cranial challenge with viable GL261 cells.

Although prophylactic vaccination is valuable for the analysis of molecular mechanisms, it does not reflect the actual clinical situation when patients are therapeutically vaccinated. Therefore, we tested the effectiveness of DC-GL261_PS-PDT vaccines in the therapeutic orthotopic mouse model (Suppl. Fig. 3A). We found that four consecutive DC-GL261_PS-PDT or DC-GL261_MTX vaccine injections significantly increased the median survival of glioma-inoculated mice by about 38% (37 days versus 51 days, p < 0.02) and also resulted in about 66% more long-term cured survivors compared to mice injected with PBS (Suppl. Fig. 3B). This finding was confirmed by analysis of neurological scores, ex vivo MRI and histological analysis (Suppl. Fig. 3C–F). To further examine the adaptive immune response induced in the therapeutic setting by DC-GL261_PS-PDT vaccines, we performed immune cell phenotyping of the isolated draining lymph nodes of vaccinated mice. Interestingly, we found that the draining lymph nodes of mice therapeutically vaccinated with DC-GL261_PS-PDT contained a significantly increased number of CD8^+^T cells compared to the control while the number of DCs and macrophages remain unchanged (Suppl. Fig. 3G).

### Glioma cells undergoing immunogenic cell death after PS-PDT induce a Th17 signature in DCs

**Fig. 4:**
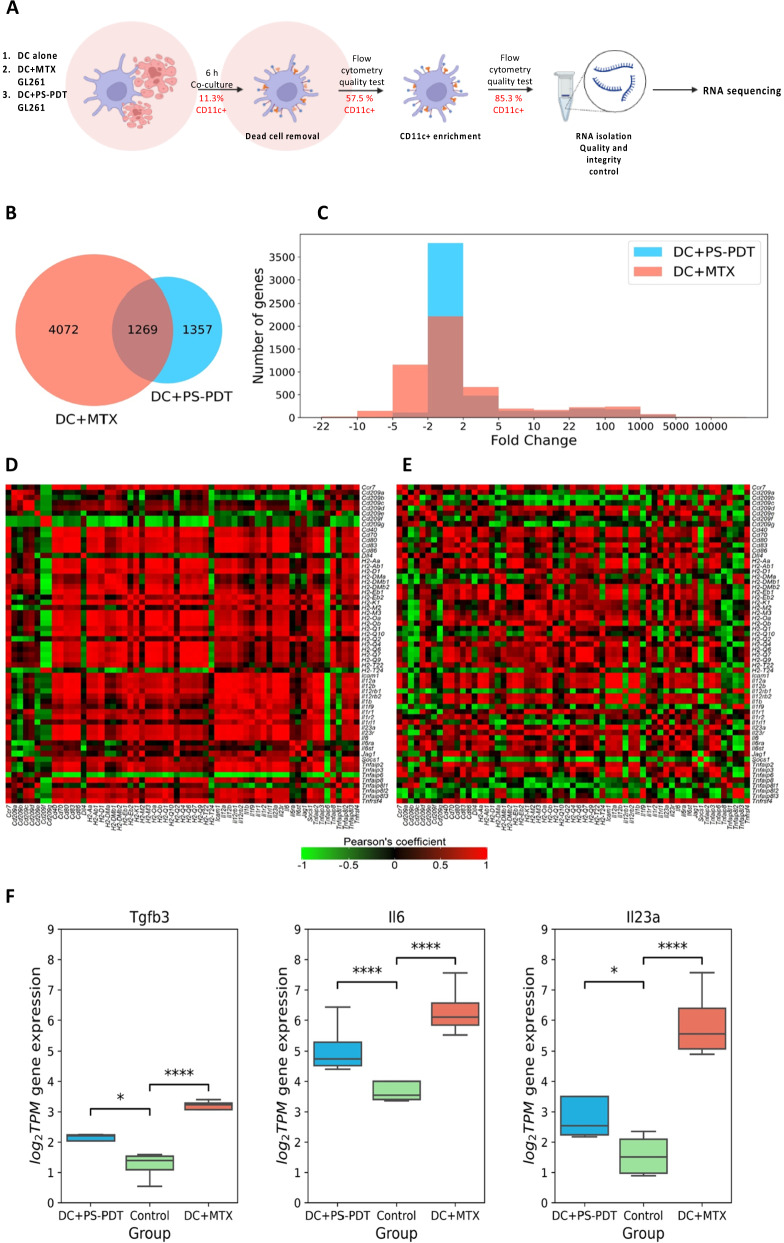
RNA sequencing expression analysis of bone marrow-derived dendritic cells (DCs) co-cultured with dying/dead GL261 cells. **A** DCs were co-cultured with GL261 cells treated with 2.0 µM MTX, or loaded with 1.4 µM photosens and exposed to PDT (PS-PDT) for 6 h. The DCs were depleted of dead cells and CD11-positive cells were purified as described in Materials and Methods and in Efimova et al. [84]. The total RNA extracted from the purified DCs was subjected to RNA-seq analysis. **B** Venn diagram showing the total number of genes that were differentially expressed more than twofold (|Fold Change| ≥ 2; adjusted *p*-value < 0.05, base Mean > 100) in the PS-PDT and MTX groups compared to controls. **C** Histograms with fold change in expression level of differentially expressed genes (adjusted *p*-value < 0.05, base Mean > 100) between the experimental groups (PS-PDT, MTX) and control. **D**, **E** Gene-gene correlation matrices. Pearson-correlation matrix of 61 marker genes of DC maturation in samples under the action of PS-PDT (**D**) and samples under the action of MTX (**E**). Each colored square within the figure illustrates the correlation between two genes. Red indicates a very strong positive correlation, black no correlation, and green a very strong negative correlation. **F** Box plots showing the expression of DC genes the products of which activate a Th17 response. The Tgfb3, Il6, and Il23a genes were strongly expressed in the PS-PDT and MTX groups compared with the control group. The *x*-axis of the plot represents different groups: PS-PDT, MTX, and control. The *y*-axis shows the expression data after log_2_(TPM + 1) transformation. Statistical significance analysis was performed using the Wald test from DESeq2. Adjusted *p*-value using Benjamini–Hochberg mode < 0.05 was considered statistically significant. *p < 0.05, **p < 0.01, ***p < 0.001, ****p < 0.0001. TPM transcripts per million.

To identify the pathways induced in DC vaccines after in vitro coculture with dying GL261_PS-PDT or GL261_MTX (positive control), we sequenced the RNA of bone-marrow-derived DCs. Before sequencing, we performed a two-step enrichment for DCs (Fig. 4A). Flow cytometry showed that this resulted in the enrichment of DCs from 11.3% to 85.3% of CD11c^+^ cells.

RNA sequencing of the DCs after 6 h of co-culture with glioma GL261 cells treated with either PS-PDT or MTX indicated distinct transcriptional changes (Fig. 4B, C). By comparing the cell sequencing results of GL261_PS-PDT with those of control DCs, we identified 1357 differentially expressed genes (1242 up and 115 down) (Fig. 4B, C). Pathway enrichment analysis revealed that GL261_PS-PDT cells altered their gene expression programs linked to cellular processes, biological regulation, metabolic processes, response to stimuli, signaling, developmental processes, multicellular organismal processes, and immune system processes (Suppl. Fig. 4A). Our positive control group, DCs cocultured with glioma GL261 cells treated with MTX, a well-known ICD inducer [19, 30], had 4072 differentially expressed genes compared with control DCs (2354 up and 1718 down, Fig. 4B, C). We also identified 1269 genes common between the PS-PDT and MTX groups (among them 1181 simultaneously up, 87 simultaneously down and 1 gene with changes in opposite directions). In addition, we analyzed markers of DC maturation, including exogenous signals (cytokines, chemokines) and ligands on the surface of DCs, and found that 61 genes were responsible for the expression of these molecules (Fig. 4D, E). Pearson correlation matrices showed that though both PS-PDT and MTX triggered ICD, they induced quite different expression profiles of the selected genes (Fig. 4D, E).

Next, to identify the immunogenic signature triggered in DCs by dying GL261_PS-PDT cells, we analyzed the marker genes responsible for T cell differentiation (Th1, CTL, Th17, and Treg). We checked whether these marker genes are among the differentially expressed genes. Remarkably, we found that DCs cocultured with GL261_PS-PDT cells showed high expression levels of genes that activate Th17 cells (Fig. 4F). The Tgfb3, Il6, and Il23a genes were strongly expressed in DCs cocultured with dying glioma cells pulsed with PS-PDT or with MTX (positive control) compared with the control group. At the same time, the marker genes of Th1, CTL, and Treg cells were not differentially expressed. These data suggest that the immunogenicity of the ICD-based DC vaccine is associated with a Th17 signature in DCs.

### Blocking RORγt reduced the protection by DC vaccines loaded with DC-GL261_PS-PDT cells in the orthotopic glioma mouse model

**Fig. 5:**
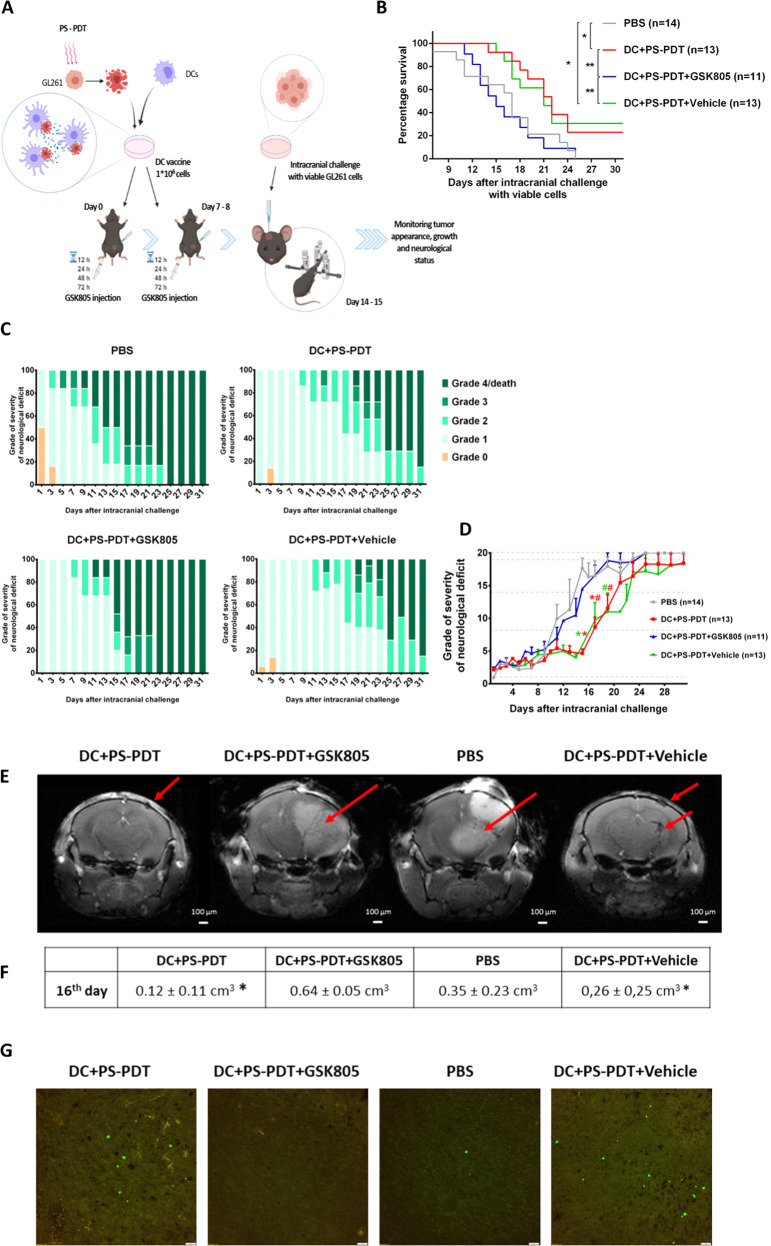
The efficacy of ICD-based DC vaccines depends on RORγt signaling. **A** Mice received DC vaccines loaded with PS-PDT on days 0 and 7. After each vaccination, the mice received four intraperitoneal injections of the RORγt inhibitor (GSK805, 10 mg/kg) or vehicle (control) 12, 24, 48, and 72 h postvaccination, or were injected with PBS. In addition, a control group received PBS instead of a vaccine. Seven days after the last vaccination with the DC-based vaccine, the mice were intracranially injected with viable GL261 cells. B The curve represents survival of the mice for up to 30 days after the intracranial inoculation of tumor cells. *P* < 0.02, Mantel-Cox logarithmic test (*n* = 13–14). **C** Analysis of the neurological status of mice for up to day 31 after the intracranial tumor inoculation. The percentage of mice after intracranial tumor inoculation showing different degrees of neurological alterations (grades 0–4) in the different groups is shown. **D** The temporal progression of neurological deficits in mice treated as described in (**A**) is shown for each group (*n* = 11–14). *p < 0.01, a statistically significant difference from the PBS group, ^#^*p* < 0.01, a statistically significant difference from the DC + PS-PDT + GSK805 group; Wilcoxon test. **E** Representative T1-tomograms of layer-by-layer frontal brain sections on day 16. The tumor mass is indicated by red arrows. **F** Tumor volume was obtained by analysis of diffusion-weighted MRI images; *n* ≥ 6 per group. **G** The sagittal brain sections of mice treated with DC + PS-PDT or DC + PS-PDT in the presence of GSK805 or treated with vehicle or PBS. The sections stained with anti-IL-17 antibodies (green) demonstrated the presence of IL-17^+^ cells in the brain of mice treated with DC + PS-PDT, but these cells were not present in the brains of mice treated with DC + PS-PDT in the presence of GSK805. Scale bars 20 μm.

Next, we used the orthotopic murine model to validate the role of Th17 cells in the anti-glioma protective immunity induced by the DC-GL261_PS-PDT vaccine. To that end, we blocked RORγt, a transcription factor regulating the expression of the pro-inflammatory cytokine IL-17 in human Th17 cells [34, 35]. We used a potent RORγt inhibitor (GSK805) that can penetrate into the central nervous system [36, 37]. GSK805 was intraperitoneally injected 12, 24, 48, and 72 h after intraperitoneal injection of the DC-GL261_PS-PDT vaccine (Fig. 5A). In mice vaccinated with the DC-GL261_PS-PDT vaccine, treatment with 10 mg/kg of the RORγt inhibitor (GSK805) significantly decreased median survival (Fig. 5B). In parallel with this decrease in overall survival, GSK805 also resulted in more noticeable clinical manifestations and earlier onset of symptoms (Fig. 5C, D). To support these data, we non-invasively monitored the mice by using MRI imaging (Fig. 5E, F). The group of mice treated with the RORγt inhibitor showed disguised glioma formation that altered ventricular morphology, deformed the cerebral cortex, and deepened the tumor lesion towards the optic nerve, resulting in exophthalmos. On the other hand, almost none of the mice vaccinated with the DC-GL261_PS-PDT vaccine showed noticeable glioma masses at the site of inoculation and all of them retained better brain morphology. Further, we observed by immunohistochemistry infiltration of IL-17^+^ cells in the brain after vaccination with the DC-GL261_PS-PDT vaccine (Fig. 5G). Moreover, the depletion of IL-17^+^ cells in the brains of mice treated with DC-GL261_PS-PDT and GSK805 was confirmed by immunohistochemistry (Fig. 5G). These data demonstrate that pharmacological inhibition of RORγt significantly reduces the immunogenic potential of the DC-GL261_PS-PDT vaccine. Collectively, these results suggest the importance of Th17 cell responses for efficient glioma DC-based therapy.

### Relationship between the Th17-associated genetic signature and patient survival

It has been proposed that the presence of specific T-lymphocyte subsets and the absence of immunosuppressive cells is associated with improved prognosis in cancer patients and can yield information relevant to the prediction of treatment response and various other pharmacodynamic parameters [38]. Therefore, we studied the clinical prognostic potential of the Th17 gene signature observed in the DC-GL261_PS-PDT vaccine. To that end, we analyzed a Th17-based immune contexture in patients with low-grade glioma (LGG) by making use of the very large, standardized and publicly available cohort of 508 LGG patients from The Cancer Genome Atlas (TCGA) [39]. The lymphocyte subtype-specific mRNA signature for Th17 cells is available from a previous study [40]. This mRNA signature was analyzed in the TCGA-LGG dataset to select a cluster of genes within this signature showing strong collective co-expression (so-called “metagene”) [14, 41] centered on the standard/specific Th17 cell marker (IL17A) [42]. To identify the LGG-specific Th17 cell-associated metagene, we calculated the co-expression of genes in the signature (correlation matrix) (Suppl. Fig. 4B). The correlation matrix was subjected to unsupervised hierarchical clustering with Euclidean distance measurement and average linkage clustering. Then, in the TCGA-LGG cohort, we calculated the prognostic impact of the expression of the Th17 metagene on overall patient survival, dividing patients into groups with low and high metagene expression by the 75th percentile. In addition, we calculated the percent difference in median survival (%Δ*MS*) between the high-expression and low-expression groups. Though the difference in overall survival was not statistically significant between the groups with high and low expression of the Th17 metagene (log-rank *p*-value > 0.05), a positive %∆MS of +10% indicates a possible trend that the strong expression of the Th17 metagene is associated with prolonged overall survival (Suppl. Fig. 4C).

These data are in line with our in vitro RNA-seq data, where glioma undergoing ICD after PS-PDT induced a significantly stronger Th17 signature in murine DCs as compared to control (Fig. 4F) and provided support for the effect of the pharmacological inhibition of RORγt on the immunogenic potential of the DC-GL261_PS-PDT vaccine (Fig. 5B–F) and the depletion of IL-17^+^ CD4^+^ T cells in the brains of mice treated with DC-GL261_PS-PDT and GSK805 (Fig. 5G). Collectively, these results suggest an important role for Th17 cell responses in the efficacy of the DC-based immunotherapy of glioma.

### Identification of the four-gene signature associated with overall survival of glioma patients

To identify the genes associated with the survival of patients with glioma, we analyzed the transcriptome data from the TCGA-LGG dataset. The differential expression of 249 genes was statistically significant (|Fold Change| ≥ 2, adjusted *p*-value < 0.05, base Mean > 50) between the DEAD and ALIVE groups of patients; 236 genes were upregulated and 13 were downregulated in the DEAD group relative to ALIVE patients. Among the genes found, we identified 158 genes that were differentially expressed (adjusted *p*-value < 0.05, base Mean > 50) in ALIVE TCGA-LGG patients compared to CONTROL patients, of which 119 genes were upregulated and 39 were downregulated.

**Fig. 6:**
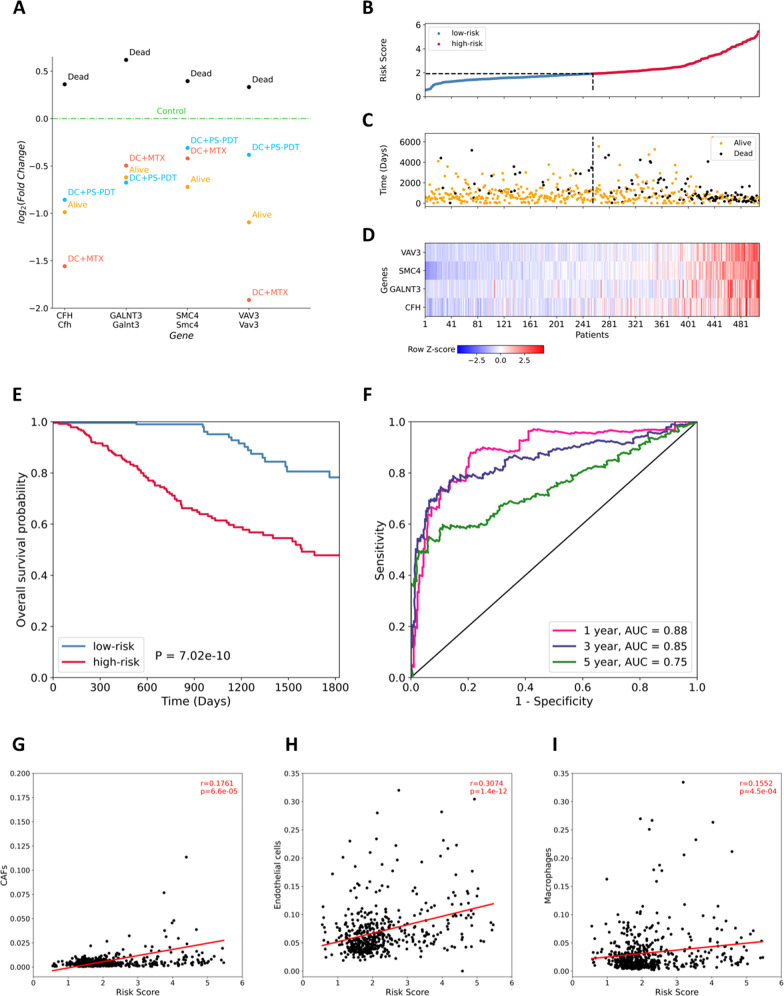
Four-gene prognostic signature and its relationship with overall survival in the TCGA-LGG dataset. **A** Plot of log_2_ Fold Change expression of the genes from the four-gene prognostic model. For each gene, values of log_2_FC are presented for the DEAD/ALIVE patient groups and for the PS-PDT/MTX groups *versus* the corresponding control groups. The conditional control level is shown with a green dash-dotted line. **B** The risk score of each LGG patient. The median risk score for categorizing patients into low-risk (blue) or high-risk (red) groups is 1.92. **C** The time to death of dead patients (black) and time until last follow-up of live patients (orange). **D** Heatmap of gene expression profiles of the four prognostic genes. **E** Kaplan–Meier survival analysis of high-risk *versus* low-risk groups. **F** Time-dependent receiver operating characteristic curve analysis of the four-gene predictive model. **G**–**I** Correlation between the four-gene prognostic signature for TCGA-LGG and the infiltration of immune and cancer cell subtypes: CAFs (**G**), endothelial cells (**H**) and macrophages (**I**).

Next, 158 genes were matched with 5135 differentially expressed genes (adjusted *p*-value < 0.05, base Mean > 100) from our RNA-seq data of DCs cocultured with glioma GL261 cells treated with PS-PDT or MTX (Fig. 4). We identified five genes (CFH, CYP1B1, GALNT3, SMC4, and VAV3) the expression of which changed in the in vitro DC experiments, and they were changed in the same direction in the ALIVE patients. Next, based on log_2_(TPM + 1) normalized expression data from the TCGA-LGG dataset, we performed univariate Cox proportional hazards regression analysis for each gene. This analysis allowed us to select four prognosis-associated genes (CFH, GALNT3, SMC4, and VAV3) that were statistically significantly correlated with overall survival (*p*-value < 0.05). The change in the expression of these four prognostic genes in the DEAD/ALIVE groups of TCGA-LGG patients and in DCs co-cultured with glioma 261 cells killed by PS-PDT or MTX groups are compared to the corresponding control groups (Fig. 6A). This analysis indicates a commonality of mechanisms with a good prognosis (ALIVE patients with low expression of these genes) and with the use of PS-PDT or MTX.

Then, we used prognosis-related genes as variables in the final multivariate Cox regression model. The С-index (index of concordance) was considered an assessment of the predictive model. Its value was 0.82, which indicates that the four-gene signature can successfully predict the prognosis of patients with glioma. In our prognostic model, the risk score = (0.121232 × expression value of CFH) + (0.114831 × expression value of GALNT3) + (0.521462 × expression value of SMC4) + (0.329177 × expression value of VAV3). The coefficients of all four genes are positive, which means that patients with high expression levels of CFH, GALNT3, SMC4, and VAV3 have low survival rates. Next, the risk score for every patient based on our prognostic model was calculated and the 508 patients were separated into low-risk (*n* = 254) and high-risk (*n* = 254) subgroups based on the median risk score of 1.92.

The distribution of the TCGA-LGG patients’ risk scores (with color-coded risk level), time to death or until the last follow-up, and RNA-seq expression of the patients are shown in Fig. 6B–D. The survival (or censoring) time of patients with high-risk scores was lower than in those with low-risk scores (Fig. 6B, C). The expression of prognostic genes was higher in high-risk patients (Fig. 6B, D). A heatmap was generated using the Z-score on log_2_(TPM + 1) normalized expression value to illustrate the relative expression levels of the genes (Fig. 6D). Z-score normalization was used in addition to centering and variance stabilization. The Kaplan–Meier overall survival curves of the two groups based on the four prognostic genes were significantly different (log-rank *p*-value = 7.02e−10 < 0.05) (Fig. 6E). We also used receiver operating characteristic curve analysis to estimate the accuracy of the risk score’s prediction of the clinical outcomes of TCGA-LGG patients (Fig. 6F). The calculated risk score was most accurate in assessing the one-year prognosis of TCGA-LGG patients, with an area under the curve of 0.88. Generally, the accuracy of the prognosis signature exceeded 0.7 in the clinical outcome prediction from one to five years (area under curve of 0.85 and 0.75 after 3 and 5 years, respectively).

Next, we analyzed the correlation between the prognostic signature and the infiltration of immune cells and cancer cells in TCGA-LGG. The fraction of B cells, CD4^+^ T cells, CD8^+^ T cells, macrophages, NK cells, cancer-associated fibroblasts, and endothelial cells was predicted using the EPIC deconvolution method. Cancer-associated fibroblasts, endothelial cells, and macrophages were significantly correlated (*p*-value < 0.05) with the risk score (Fig. 6G–I), and Pearson’s correlation coefficients were 0.1761, 0.3074 and 0.1552, respectively. This indicates that the infiltration of cancer-associated fibroblasts, endothelial cells, and macrophages is positively correlated with the poor prognosis of TCGA-LGG, and an increase in the proportions of these cells is associated with an increase in the risk score. There was also a trend towards a decrease in the proportion of CD4^+^ and CD8^+^ T cells in patients with high-risk scores, but this correlation was not statistically significant (data not shown).

### Validation of the prognostic four-gene signature on overall survival in the CGGA-LGG, TCGA-GBM, and CGGA-GBM datasets

**Fig. 7:**
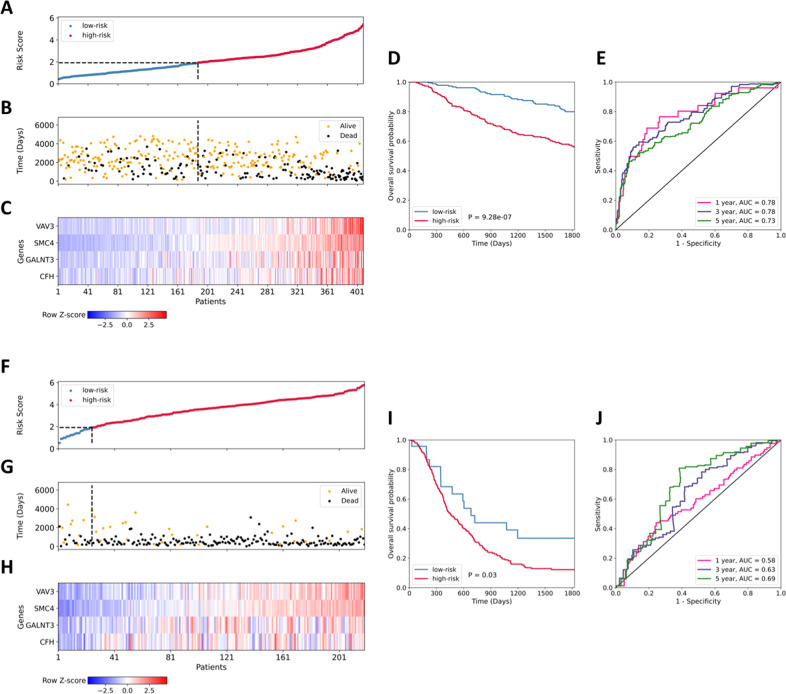
Validation of the prognostic four-gene signature for overall survival in the CGGA-LGG, TCGA-GBM and CGGA-GBM datasets. **A**–**E** Characteristics of the four-gene prognostic signature in the validation CGGA-LGG dataset. **A** The low- and high-risk scores for each CGGA-LGG patient. **B** The time to death of patients (black, DEAD) and time until last follow-up of live patients (orange, ALIVE) from the CGGA-LGG dataset. **C** Heatmap of gene expression profiles of the four prognostic genes in the CGGA-LGG dataset. **D** Kaplan–Meier survival analysis of high-risk *versus* low-risk groups in the CGGA-LGG dataset. **E** Time-dependent receiver operating characteristic curve analysis of the four-gene predictive model in the CGGA-LGG dataset. **F**–**J** Characteristics of the four-gene prognostic signature in the validation CGGA-GBM dataset. **F** The low- and high-risk scores of each CGGA-GBM patient. **G** The time to death of dead patients (black, DEAD) and time until last follow-up of live patients (orange, ALIVE) from the CGGA-GBM dataset. **H** Heatmap of gene expression profiles of the four prognostic genes in the CGGA-GBM dataset. **I** Kaplan–Meier survival analysis of high-risk *versus* low-risk groups in the CGGA-GBM dataset. **J** Time-dependent receiver operating characteristic curve analysis of the four-gene predictive model in the CGGA-GBM dataset.

To determine whether the four-gene prognostic signature had similar prognostic value in different cohorts, we first used the CGGA-LGG dataset for validation. The risk score of each patient was calculated according to the risk score formula derived from the training TCGA-LGG dataset. The 408 patients in the validation CGGA-LGG set were separated into low-risk (*n* = 186) and high-risk (*n* = 222) subgroups based on the training set cutoff value of 1.92. Next, the distribution of risk score, survival status, and the heatmap of prognostic gene expression in the CGGA-LGG dataset were analyzed (Fig. 7A–C). Consistent with the results of the TCGA-LGG dataset, the Kaplan–Meier survival curves and log-rank test (*p*-value = 9.28e−07) revealed a significant difference in overall survival between the low- and high-risk groups in the CGGA-LGG dataset (Fig. 7D). Receiver operating characteristic curve analysis of the four-gene model was conducted: the values of area under curve were 0.78, 0.78, and 0.73, respectively, for predicting 1-, 3-, and 5-year survival rates (Fig. 7E).

In addition, the performance of the prognostic model built for patients with LGG was tested on patients with glioblastoma multiforme (GBM). To do this, we repeated the validation procedure for the TCGA-GBM and CGGA-GBM datasets. When dividing TCGA-GBM patients into low- and high-risk groups according to the cutoff value, 149 of the 151 patients were in the high-risk group. Receiver operating characteristic curve analysis of the four-gene model revealed that the values for the TCGA-GBM dataset of area under curve were 0.9 for predicting 5-year survival rate. A similar trend, but not as extreme, was observed in the CGGA-GBM dataset: there were more patients in the high-risk group (*n* = 195) than in the low-risk group (*n* = 23) (Fig. 7F–H). These results were expected, since glioblastoma is a more aggressive tumor than glioma and the number of dead patients in the GBM datasets is greater than the number of those alive. Consistent with the results of the low-grade glioma datasets, patients in the high-risk group had a poorer prognosis in the CGGA-GBM dataset, with log-rank *p*-value = 0.03 (Fig. 7I). The 1-, 3- and 5-year area under curve were 0.58, 0.63 and 0.69, respectively (Fig. 7J).

## Discussion

**Fig. 8:**
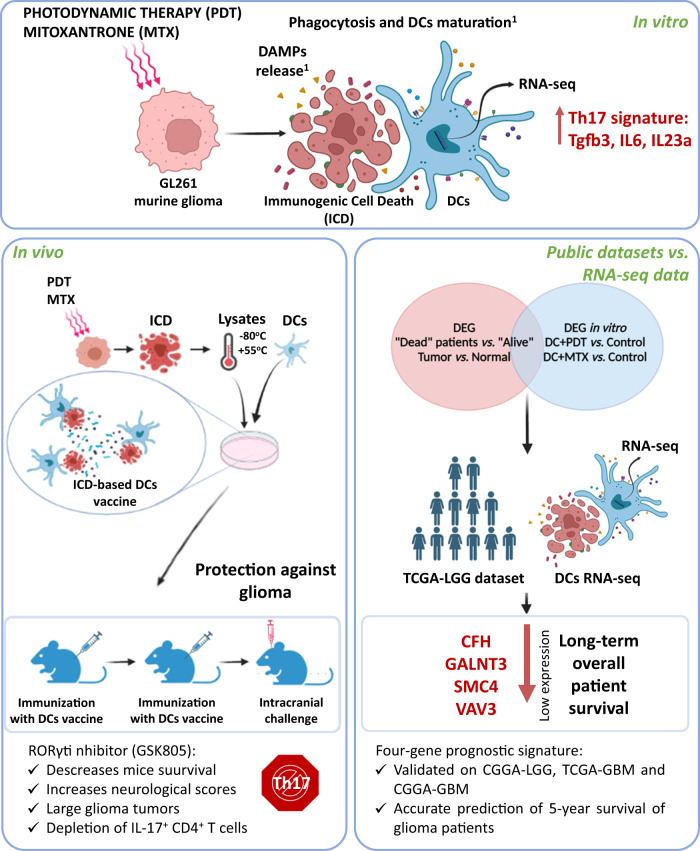
DC vaccines loaded with glioma cells killed by PS-PDT induce Th17 anti-tumor immunity and provide a four-gene signature for glioma prognosis (graphical abstract). We developed ICD-based DC vaccines and demonstrated that they induce significant protective immunity against glioma in the orthotopic model. To investigate the molecular mechanism of DC vaccines loaded with glioma cells undergoing ICD, we performed a comparative analysis of their transcriptional levels in two ICD-inducing modalities (PS-PDT and MTX) and we analyzed the marker genes responsible for T cells differentiation (Th1, CTL, Th17, and Treg). This study revealed that the expression of DC genes the products of which (Tgfb3, Il6, and Il23a) activate Th17 cells were expressed at high levels in DCs cocultured with dying glioma cells treated with PS-PDT or MTX compared to control. Notably, blocking RORγt reduced the protection of DC vaccines loaded with dying glioma cells treated with PS-PDT in the orthotopic glioma model. Finally, by matching differentially expressed genes (DEG, between “ALIVE” and “CONTROL” patients in TCGA-LGG datasets) from our RNA-seq data of DCs cocultured with glioma GL261 cells treated with PS-PDT or MTX, we established a predictive model based on the four-gene signature (CFH, GALNT3, SMC4, and VAV3). Application of this signature to the TCGA-LGG dataset predicted the patients’ overall survival. When it was validated on the overall survival of the CGGA-LGG, TCGA-GBM, and CGGA-GBM datasets, it accurately predicted the five-year survival rates. In conclusion, in this study we have shown that DC vaccines loaded with glioma cells killed by photodynamic therapy induce Th17 anti-tumor immunity and provide a four-gene signature for glioma prognosis. These findings open attractive prospects for improving glioma therapy by employing ICD and PS-PDT-based DC vaccines to induce Th17 immunity and to using the prognostic model to predict the overall survival of glioma patients. ^1^Of note, in the previous study we demonstrated that dying/dead GL261 cells treated with PS-PDT undergo typical hallmarks of ICD such as exposure on their surface calreticulin and release DAMPs such as ATP and HMGB1 [20]. Importantly dying/dead GL261 treated with PS-PDT induce efficient activation and maturation of DCs in vitro [20] justifying their use as a vaccine in the current study.

In this study, we found that DC vaccines primed with glioma cells undergoing ICD after PS-PDT protect mice not only against challenge with viable glioma GL261 cells in several orthotopic glioma models in the prophylactic mode but are also effective in the curative setting, which most closely resembles the clinical setting. Moreover, by using RNA-seq analysis, we found that while glioma cells are undergoing ICD after PS-PDT, they induce a typical Th17 signature in the DC vaccines and that these vaccines were highly effective in protecting mice against gliomas (Fig. 8). Furthermore, the effect of these DC vaccines was significantly reduced when a specific RORγt inhibitor was used, confirming that a Th17-induced anti-tumor immune response is required for the efficacy of these DC vaccines in the murine orthotopic glioma model. Furthermore, by comparing the transcriptome program of the ICD-based DC vaccine with the transcriptome data from the TCGA-LGG patients, we identified a four-gene signature (CFH, GALNT3, SMC4, and VAV3) that is strongly associated with overall survival of glioma patients, and we validated it on the CGGA-LGG, TCGA-GBM, and CGGA-GBM datasets (Fig. 8).

Immunotherapy has emerged as a standard of care and the first-line treatment for several cancer types, including glioma, mainly due to the discovery of immune checkpoint inhibitors and their significant clinical impact [43]. One of the promising concepts of cancer immunotherapy relies on the induction of ICD, which is on the one hand characterized by adjuvanticity and emission of DAMPs and cytokines, leading to activation of anti-tumor immunity, and on the other hand dictated by the antigenicity of dying cancer cells, which is defined by the level of tumor (neo)antigens [23, 44, 45]. Adjuvanticity and antigenicity of dying cancer cells are required for generation of anti-tumor immunity and long-lasting immune memory, which is required for lifelong protection of patients. In recent years, several promising ICD-inducing therapeutic modalities have been introduced, including therapeutic strategies based on PDT, which is effective for certain types of cancer. PDT involves the use of a photosensitizing agent and photoexciting light, which, in the presence of molecular oxygen, generate singlet oxygen and other cytotoxic oxidants that trigger ICD [26, 28, 46]. Importantly, the potential of ICD in cancer therapy has been well-established [23–25, 44, 45], and most studies have focused on the induction of ICD in murine heterotopic cancer models [14, 19, 27, 29, 47]. However, in this study, we developed DC vaccines primed with glioma cells undergoing ICD after PS-PDT for glioma therapy. It is noteworthy that several different methods are available for designing patient-derived cancer cell vaccines. Two widely used methods are the use of dying cancer cells [14, 19, 27, 30, 47] and DCs primed with tumor cell lysates [14, 48]. In our work, we subjected glioma cells to PS-PDT, leading to the induction of the typical features of ICD, followed by preparing tumor cell lysates and priming DCs with them. We had already characterized PS-PDT in our previous work [20] and showed that glioma GL261 cells subjected to PS-PDT undergo ICD with the emission of the three major DAMPs (CRT, ATP, and HMGB1), thereby inducing activation and maturation of DCs in vitro [20]. In our current study, we developed in orthotopic glioma models a novel immunotherapy based on a DC vaccine pulsed with GL261 glioma cells treated with PS-PDT.

Importantly, whole glioma tumor cells are often lysed by several F/T cycles, which leads to unregulated cell death known as accidental necrosis, which has limited immunogenic potential [19, 20, 27]. In our current study, we used two different orthotopic glioma prophylactic mouse models: subcutaneous vaccination with glioma GL261 cells undergoing ICD (Fig. 2) and intraperitoneal vaccination with DC vaccines pulsed in vitro with glioma GL261 cells killed by PS-PDT or F/T, or treated with MTX as a positive control (Fig. 3). The results demonstrate that the ICD induced in glioma GL261 cells killed by PS-PDT provided considerable survival benefits against challenge with viable GL261 cells in both mouse models as compared to vaccination with glioma cells after F/T. Notably, the DC-GL261_PS-PDT vaccine increased the median survival time by more than 12% as compared to the F/T group. These data confirm the previously reported observation of the non-immunogenicity of cancer cells undergoing accidental necrosis [19, 20, 27, 29]. Interestingly, DC-GL261_PS-PDT was also effective for treating existing glioma in mice in the curative mode as well (Suppl. Fig. 3). Moreover, we found that PS-PDT induced a mixed type of regulated cell death in GL261 cells with features of both apoptosis and ferroptosis. Induction of cell death by PDT can also be beneficial because PDT can work synergistically with ferroptosis to provide a source of reactive oxygen species for the Fenton reaction [28]. The dose of photosensitizer used to trigger cell death must also be considered because there is a non-linear relationship between photosensitizer concentration and the PDT-induced antitumor immune response [49].

To investigate the efficacy of DC vaccines further and to unravel the immunogenic signature triggered in the DCs by dying GL261_PS-PDT cells, we performed RNA-seq analysis of DCs co-incubated with dying glioma GL261 cells triggered by PS-PDT or MTX treatment. Importantly, we found significantly different expression levels of the Tgfb-3, IL-6, and IL-23a genes in the DCs pulsed with dying cells undergoing ICD after PS-PDT or MTX treatment as compared to control untreated DCs. It is known that the Th17 genetic signature is specific to a Th17 immune cell response and contributes to the steering of this response in mice and in humans [50]. Th17 cells have been identified as an independent subtype of inflammatory T cells with an IL-17 and transcription factor RORγt profile [51]. Of note, naive CD4^+^ T cells can be induced to differentiate, depending on the local cytokine milieu, towards a T helper-1 (Th1), Th2, Th17, or regulatory T cell phenotype with unique signaling pathways and expression of specific transcription factors [52]. Thus, our RNA-seq data clearly point to the activation of the Th17 signature in the DC vaccines after their coculture with GL261_PS-PDT cells. We previously reported that glioma GL261 cells subjected to PS-PDT and co-cultured with DCs induced the production of IL-6 [20], indirectly supporting our current RNA-seq data (Fig. 4). It is also known that dying cancer cells undergoing ICD induce a typical Th1 signature in vitro and in vivo [14, 19]. But our data provide more specific direct experimental evidence pointing to the ability of glioma undergoing ICD to induce a Th17 molecular signature in antigen-presenting cells (i.e., DCs). Furthermore, in the prophylactic orthotopic glioma model, co-injection of a specific inhibitor (GSK805) of RORγt, the transcription factor of the Th17 response, significantly reduced the protective effects of the DC vaccine based on ICD and PS-PDT. Moreover, we found that TCGA-LGG patients have +10% %∆MS, which suggests that the Th17 metagene might be associated with prolonged overall survival (Suppl. Fig. 4B, C), confirming a previous report on the relationship between high Th17 metagene expression and longer survival of patients with GBM [14]. Interestingly, when patients with mutated isocitrate dehydrogenase 1 (IDH1), a molecularly distinct subtype of diffuse glioma, were vaccinated with IDH1(R132H)-specific peptide vaccine, they showed production of tumor necrosis factor (TNF), interferon-γ (IFNγ), and IL-17 upon in vitro re-stimulation of peripheral IDH1-vaccine-induced T cells with IDH1(R132H), which indicates the involvement of Th1 and Th17 subtypes of Th cells [53]. These results also indirectly support our current data obtained in a prophylactic orthotopic glioma model in which a specific inhibitor of RORγt was also injected. The results point to a promising strategy for glioma therapy by employing ICD and PS-PDT-based DC vaccines to induce Th17 immunity. However, it is important to stress that even though multiple types of IL-17-producing cells are found in the tumor bed in mouse models and in humans, their role in tumor progression remains controversial [54]. It is noteworthy that our findings are in striking contrast to the previously reported role of Th17 and IL-17 in glioma promotion, where a direct correlation between two-year progression-free survival and low incidence of IL-17 producing cells was reported [55]. However, the molecular mechanism of IL-17-mediated glioma progression was not shown in that study. In another recent study, the CD8^+^ and CD4^+^ tumor-infiltrating lymphocyte compartment was characterized in depth. The results pointed to a pronounced Th17 commitment of CD4^+^ tumor-infiltrating lymphocytes in untreated GBM patients [56]. Although the authors proposed that exaggerated Th17 responses in the GBM bed may create a dominant-negative environment for productive Th1 and CTL responses blocking adaptive antitumor immunity, a direct link between exaggerated Th17 responses and survival of GBM patients has been not identified. Indeed, the brain tumor microenvironment varies greatly during its progression from early to late disease, among different tumor types, among individuals with the same diagnosis, and in non-neoplastic cell types and cell states [57]. In this regard, it has been shown that myeloid cells in GBM tissue are the dominant immune cell type [57–59] and that GBM has much fewer lymphocytes, and in particular T cells such as CD4^+^ and CD8^+^ T cells, which represent about 10 and 7%, respectively, of the immune cell infiltrate in GBM [56]. Therefore, the underlying molecular and cellular mechanisms of the role of IL17 in cancer are still not completely understood. Many interesting and challenging findings are expected.

Our study allowed us to identify the four-gene signature (CFH, GALNT3, SMC4, and VAV3) associated with the overall survival of glioma TCGA-LGG patients. Currently, there are various prognostic models to predict the survival of LGG patients. In our study, we looked at genes that show commensurate expression changes in a group of live LGG patients and in the PS-PDT and MTX groups in our in vitro experiments. Although PS-PDT and MTX have different effects on the activation of immune system genes, both have pronounced positive effects in modeling the oncological process. Using TCGA-LGG data, we show that despite the differences in mechanisms, some genes could have significant prognostic value in distinguishing good from poor prognosis. Based on the immune cell deconvolution method, we found a significant correlation between the four-gene prognostic signature and the infiltration of immune and cancer cell subtypes. The results demonstrate that patients with higher infiltration levels of cancer-associated fibroblasts, endothelial cells and macrophages have a poor prognosis. Of note, these four genes that constitute the prognostic model have been shown to have the following associations: (i) high expression level of Complement Factor H (CFH) has been associated with the progression of cutaneous squamous cell carcinoma [60]; (ii) GALNT3 (polypeptide N-acetylgalactosaminyltransferase 3) has been linked to neuroblastoma [61]; (iii) strong expression of structural maintenance of chromosomes 4 (SMC4) promotes an aggressive phenotype in glioma cells [62]; (iv) depletion of Vav3 (guanine nucleotide exchange factor) by siRNA oligonucleotides suppresses GBM cell migration and invasion [63]. These findings reinforce the functional relevance of this four-gene prognostic signature.

Altogether, our findings point to the importance of the Th17 signature as a prognostic marker and to its positive therapeutic impact in glioma therapy based on DC vaccines pulsed with dying cancer cells undergoing ICD triggered by PS-PDT (Fig. 8). Considering the wide GBM heterogeneity and plasticity, which result in the lack of “quality” neoantigens, DC vaccines pulsed with dying cancer cells undergoing ICD triggered by PS-PDT may represent an attractive approach for producing whole-tumor derived immunogenic neoantigens for effective glioma therapy. Moreover, a key interpretation of our new four-gene signature model, which demonstrated a strong predictive power in both the training and validation cohorts, may help to develop novel and testable prognostic and therapeutic opportunities for glioma patients.

## Materials and methods

### Cell lines

Murine glioma GL261 cells were cultured at 37 °C under 5% CO_2_ in Dulbecco’s Modified Eagle’s Medium (DMEM) containing 4.5 g/L glucose and supplemented with 2 mM glutamine, 100 µM sodium pyruvate, 100 units/ml penicillin, 100 µg/L streptomycin and 10% fetal bovine serum, all purchased from Thermo Fisher Scientific. GL261 cells were kindly provided by Prof. P. Agostinis (Laboratory of Cell Death Research & Therapy, Department of Cellular and Molecular Medicine, KU Leuven, Leuven, Belgium).

### Quantification of cell death induction by PS-PDT and MTX and analysis of cell death inhibitors

Cell death was induced by photosens (PS)-based PDT or mitoxantrone (MTX, Sigma Aldrich). For PS-PDT, GL261 cells were first incubated with 1.4 µM PS in serum-free DMEM for 4 h and then irradiated with a light dose of 20 J/cm^2^ in photosensitizer-free media. After PDT, the cells were cultured in complete DMEM for 24 h. For MTX induction, the cells were cultured in full medium with 2.0 µM MTX for 24 h. Cells loaded with PS or MTX were handled in the dark or in subdued light. Control cells were cultured in the same conditions but without agents or PDT. Accidental necrosis in glioma GL261 cells was induced by three cycles of freezing (–80 °C) and thawing (37 °C).

The following cell death inhibitors were used: the pan-caspase inhibitor carbobenzoxy-valyl-alanyl-aspartyl-[O-methyl]-fluoromethylketone (zVAD-fmk, 25 μM, Sigma-Aldrich), the RIPK1 inhibitor necrostatin-1s (Nec-1s, 20 μM, Abcam), the inhibitor of reactive oxygen species and lipid peroxidation ferropstatin-1 (Fer-1, 1 μM, Sigma-Aldrich) and the iron chelator, deferoxamine (DFO, 10 μM, Sigma-Aldrich). The cell death inhibitors were added together with the corresponding reagent and the cells were incubated for 4 h in serum-free DMEM with PS and 24 h in complete DMEM with MTX. Before PDT, the medium was replaced with complete DMEM containing the respective cell death inhibitor, the cells were irradiated with light at 20 J/cm^2^, and then they were incubated for 24 h.

MTT assay (AlfaAesar) was performed using 3-(4,5-dimethylthiazol-2-yl)-2,5-diphenyl-2H-tetrazolium bromide according to the manufacturer’s instructions and the optical density was measured at 570 nm.

### Generation of bone-marrow-derived dendritic cells (DCs)

Bone marrow was isolated from tibias and femurs in RPMI medium (GIBCO) supplemented with 5% heat-inactivated fetal calf serum, 20 ng/ml murine GM-CSF (UGent-IRC-VIB Protein Core Facility), 1% L-glutamine, and 50 μM 2-mercapthoethanol. The bone marrow was suctioned with a 25 G needle (0.5 × 25 mm), resuspended, and coarse debris was filtered through a Cell Strainer 70 μm (Falcon). The suspension was cleared of erythrocytes with a lysing solution. The cells were grown for up to 10 days. Fresh culture medium was added on day 3, and on days 6 and 9 the medium was fully refreshed.

### Phagocytosis assay

Target glioma GL261 cells were labeled with 1 μM CellTracker Green CMFDA (Molecular Probes) in serum-free DMEM for 30 min and then induced by MTX as described above. For the PDT group, GL261 cells were loaded with 1.4 µM PS in serum-free DMEM for 4 h and then either left untreated or cell death was induced by PDT as described above. The cells were collected, washed, and co-cultured with bone marrow-derived dendritic cells (DCs) in ratios of 1:2 or 1:5 for 2 h. Next, the co-cultured cells were harvested, incubated with a mouse Fc block CD16/CD32 (ThermoFisherScientific), stained with PE-Cy7-anti-CD11c (BD PharMingen, 561022), and finally analyzed by flow cytometry on a CytoFlex (Beckman Coulter). Analysis was performed by using CytExpert software. True uptake of dead cells labeled with 5-chloromethylfluorescein diacetate (CMFDA) by DCs was determined by using a gating strategy that allows analysis only of singlets that are identified as CD11c^+^ CMFDA double-positive cells.

### DCs enrichment after coculture with glioma GL261 cells for RNA isolation

DCs isolated from four C57BL/6J mice were used for each experimental group. DCs were cocultured for 6 h with dying/dead glioma GL261 cells in a ratio of 1:5. There were three experimental groups: DCs alone (negative control) and DCs cocultured with glioma GL261 cells pulsed with PS-PDT (PS at a dose of 1.4 µM, 24 h) or with MTX (positive control, 2 µM, 24 h). For the cocultures of DCs with treated GL261 cells, additional technical replicates were included. After 6 h of coculture, the cells were harvested and washed once with DPBS. Enrichment of DCs from the coculture proceeded as follows. First, dead cells were removed with a Dead Cell Removal Kit (MACS Miltenyi Biotec). Then, the final eluate was loaded onto MS columns (MACS Miltenyi Biotec) and treated with CD11c MicroBeads UltraPure (MACS Miltenyi Biotec) to enrich for CD11c^+^ DCs. The purity of CD11c^+^ DCs was analyzed by flow cytometry on a BD FACS Canto II flow cytometer after each step. Enriched CD11c^+^ cells were snap frozen in liquid nitrogen, and total RNA was isolated using RNeasy Mini Kit (QIAGEN). The quantity and integrity of the RNA were determined with a NanoDrop 8000 spectrophotometer (Thermo Fisher) and a Fragment Analyzer 5200 system (Agilent), respectively.

### RNA-seq analysis, RNA-seq pipeline, and data quantification

The TruSeq Stranded mRNA kit (Illumina) was used to prepare a RNA-seq library according to the manufacturer’s protocol, followed by PE100 cycle sequencing on one lane of a NovaSeq 6000 S1 (Illumina). Quality assessment of the raw FASTQ files obtained after sequencing was controlled with FastQC tool v0.11.5 [64]. All the files passed quality control. Next, the BWA-MEM tool v.0.7.17 [65] was used to map raw RNA-seq reads against Ensembl *Mus musculus* GRCm39 and FeatureCounts v2.0.1 [66] to get quantification estimates at the transcript level.

### Mice experiments

Female C57BL/6J mice (7–8 weeks old) were housed in specific pathogen-free conditions. The mouse experiments were performed according to the guidelines of the local Ethics Committees of the National Research Lobachevsky State University of Nizhny Novgorod (Russia) and the Faculty of Medicine and Health Sciences of Ghent University (Belgium; ECD 22-12). The G*Power 3.1.5 software was used to determine the sample size for in vivo experiments. In some experiments, 5–14 mice per group were used without the calculation of the power.

### In vivo prophylactic tumor sub-cutaneous vaccination mouse model

Cell death was induced in GL261 cells in vitro by PS-PDT or MTX as described above. Next, the GL261 cells were collected, washed once in PBS, and re-suspended at the desired cell density in PBS. Mice were inoculated subcutaneously with 5 × 10^5^ dying GL261 cells or with PBS in the left flank. The mice were immunized once or twice with an interval of seven days. Eight days after the last vaccination, the mice were challenged subcutaneously on the opposite flank with 1 × 10^5^ live GL261 cells. Tumor growth at the challenge site was monitored using a caliper for up to four weeks after the challenge. The mice were sacrificed when the tumors became necrotic or exceeded 2000 mm^3^.

### In vivo prophylactic tumor sub-cutaneous vaccination followed by orthotopic intracranial challenge

C57BL/6 J mice were immunized subcutaneously once or twice with an interval of 7 days with GL261 cells stimulated in vitro with PS-PDT or with MTX or subjected to F/T cycles as described above. Seven days after the last vaccination, the mice were anesthetized with isoflurane (5% induction, 1.5–2% maintenance), an incision was made in the scalp, and the skull was exposed. GL261 glioma cells (20,000 cells/3 μl saline) were injected into the brains at the following coordinates: AP: –2.0 mm, ML: –2.0 mm, DV: –3.3 mm (relative to bregma). The cells were injected at a rate of 0.3 µl/min via a Hamilton syringe mounted in a motorized stereotactic injector (World Precision Instruments). After injection, the needle was left in situ for 5 min and then removed slowly. The scalp was then sewn shut and analgesia was administered (Xylanite 0.02 mg/kg) (NITA-PHARM, Russia).

### DC vaccination in an orthotopic glioma mouse model and pharmacological inhibition of RORγt

#### DC vaccines

DCs were isolated according to the protocol described above. Between days 8 and 10 of cultivation, cells were collected for co-culturing. GL261 tumor cells were stimulated as described above and incubated for 24 h. The cells were then subjected to six cycles of freezing (–80 °C) and thawing (+55 °C). Total protein in the cell lysate was measured with a commercial BCA Protein Assay Kit (Sigma-Aldrich) and a Synergy MX spectrophotometer (BioTek Instruments Inc., USA). Two mg of protein was added to a suspension of 10 × 10^6^ DCs for 90 min. To activate the DCs, they were treated with lipopolysaccharide (0.5 μg/ml) for 24 h. In some experiments, PBS or DCs co-cultured with GL261 glioma cell lysates subjected to several freeze/thaw cycles to induce accidental necrosis (without photoinduction) were used as controls.

#### Prophylactic protocol

Female C57BL/6j mice (6–8 weeks old) were injected intraperitoneally twice seven days apart with a suspension containing 1 × 10^6^ prepared DCs. Seven days after the last injection, 2 × 10^4^ viable GL261 glioma cells were injected intracranially. All animals were anesthetized with a mixture of medical oxygen and isoflurane (induction: 5%; maintenance: 2%) and immobilized in a stereotaxic frame. The injection was performed using a stereotactic device 2 mm lateral and 2 mm posterior to the bregma and 3 mm below the dura mater according to a previously described protocol [14]. The skin was sutured, and meloxicam was administered subcutaneously (1 mg/kg, 2 mg/mL) to manage post-operative pain.

To inhibit a Th17 cell response, GSK805 (10 mg/kg; InvivoChem) dissolved in 10% DMSO and 90% corn oil (Sigma Aldrich) or vehicle (10% DMSO and 90% corn oil) were administered intraperitoneally to mice 12, 24, 48, and 72 h after injection of the DC vaccine.

#### Therapeutic protocol

Female C57BL/6j mice (6–8 weeks old) were anesthetized with a mixture of medical oxygen and isoflurane (induction: 5%; maintenance: 2%) and intracranially injected with 2 × 10^4^ viable GL261 glioma cells. The injection was performed using a stereotactic device 2 mm lateral and 2 mm posterior to the bregma and 3 mm below the dura mater according to a previously described protocol [14]. The skin was sutured, and meloxicam was administered subcutaneously (1 mg/kg, 2 mg/mL) to manage post-operative pain. To prepare the DC vaccine, cell death was induced in GL261 cells in vitro by PS-PDT or MTX as described above. The mice were injected intraperitoneally with a suspension containing 1 × 10^6^ of the prepared DCs (as described above) on days 2, 6, 10, and 17 after intracranial injection of viable GL261 cells. Local (inguinal and axillary) draining lymph nodes were collected on day 37 after intracranial injection with GL261 cells. The immune cells in the draining lymph nodes were stained by anti-CD8a (eBioscience, 12-0081-81), anti-CD45 (Biolegend, 103125), mouse Fc block (eBioscience, 16-0161-85), anti-CD11b (Invitrogen, 12-0112-83) and anti-CD11c (BD Pharmingen, 561022) and analyzed on a BD FACS Canto II flow cytometer.

### Neurological status assessment

After intra-cranial inoculation with glioma Gl261 cells and/or DC vaccines, the mice were monitored three times per week and clinical symptoms were scored with a neurological deficit grading scale [14]. The dynamics of the functional state of the central nervous system was evaluated on a scale to assess the severity of neurological deficit, with modifications for mice. The scale includes several tests of motor activity, coordination, reflexes, muscle tone, ptosis, and exophthalmos. Each test was scored 2 points for no reaction, 0 for good/normal reaction, and –1 for some disturbances. The values were summed up and interpreted as severe central nervous system damage (10─20 points), moderate damage (6─9 points), or light damage (1─5 points). The neurological score was evaluated by a blinded investigator.

### Magnetic resonance imaging

To assess the dynamics of intracranial tumor growth in the prophylactic model, magnetic resonance imaging (MRI) was applied using a high-field magnetic resonance tomograph, Agilent Technologies DD2-400 9.4 T (400 MHz) with a volume coil M2M (Н1). The animals were kept under general anesthesia (0.2 mg Zoletil and 0.5 mg Xylanit, intramuscular) in a fixed position inside the magnet tunnel for 40 min. The VnmrJ program was used to obtain and process data. T1-tomograms of layer-by-layer frontal brain sections weighted by proton density were obtained using the multi gradient-echo multi slice (MGEMS) pulse sequence with the following parameters: TR = 1000 ms, TE = 1.49 ms, 6 echoes, FOV 20 × 20 mm, matrix 128 × 128 and after −256 × 256, slice thickness 1 mm, 15 slices, 17 min and 4 s scanning time.

To assess the dynamics of intracranial tumor growth in the therapeutic model, ex vivo MRI was performed on 7-T micro-MRI (PharmaScan 70/16, Bruker BioSpin, Ettlingen, Germany) as previous described [67].

### Immunohistochemical analysis in an orthotopic glioma mouse model

In the prophylactic model, the mice were terminally euthanized and perfused with sodium chloride followed by 4% formalin. The brains were dissected and embedded in paraffin, and 10-µm sections were cut. Following antigen retrieval in citric acid buffer the sections were stained overnight at +4 °C with rabbit polyclonal anti-IL-17A antibody (ab 79056, Abcam, Cambridge, UK). As secondary antibodies, goat anti-rabbit IgG conjugated to AlexaFluor488 (A11034), Invitrogen were used.

In the therapeutic model brains were rinsed three times in PBS. The brains were dissected and embedded in paraffin, and 10-µm sections were cut and stained with hematoxylin/eosin. The images were taken on a spinning disk confocal Nikon Ti2 fluorescence microscope (Nikon, Japan).

### Public datasets

The brain lower grade glioma (LGG) project dataset was downloaded from The Cancer Genome Atlas (TCGA) [39] (https://portal.gdc.cancer.gov/) in August 2022. Patients with no reported vital status, with recurrent tumor, or with an unknown survival time were excluded. There were 508 patients with the primary tumor, of whom 125 had died and the remaining 383 were alive. We used STAR-counts files containing the number of mapped reads for each gene.

We also constructed a control group of 42 healthy postmortem brain transcriptome samples. To do this, we took the gene’s count numbers in healthy samples from publicly available datasets GSE80336 [68] and GSE78936 [69] from the Gene Expression Omnibus (GEO) [70] repository (https://www.ncbi.nlm.nih.gov/geo/).

As for the validation dataset for the prognostic model, we used samples with LGG from the Chinese Glioma Genome Atlas (CGGA) [71] (http://www.cgga.org.cn/). The RNA sequencing data presented as STAR-counts of two batches (mRNAseq_325 and mRNAseq_693) and corresponding clinical information of LGG samples were combined into a single CGGA-LGG dataset containing 408 samples (164 dead and 244 alive). For additional verification, we also considered RNA-seq data of glioblastoma multiforme (GBM) from the TCGA-GBM dataset (151 samples: 122 dead, 29 alive) and CGGA-GBM dataset (218 samples: 183 dead, 35 alive), combining GBM samples from mRNAseq_325 and mRNAseq_693 CGGA batches.

The sequence of TCGA, CGGA and GEO datasets were filtered to leave only protein-coding genes. In addition, to correct for a batch effect when combining data from different batches (datasets of healthy samples or mRNAseq_693 and mRNAseq_325 batches from CGGA) the *ComBat-seq* algorithm of the *sva* v3.42.0 software [72, 73] R package was used. Finally, the expression count data were normalized by the transcripts per million (TPM) method and the normalized expression values were transformed to log_2_ values.

### Differential expression and functional annotation analysis

Differential expression analysis was conducted in R software v4.1.2 and calculated using negative binomial generalized linear modeling implemented in the DESeq2 package v1.34.0 [74]. Genes were considered differentially expressed when the *q*-value cutoff (FDR adjusted p-value using Benjamini–Hochberg mode) [75] was <0.05. To identify genes with significant differential expression, we set the following selection criteria: (i) the absolute factor of change in expression between the groups is ≥2 (|Fold change| ≥ 2); (ii) the average of the normalized count values for all samples is >100 (base Mean > 100). To identify differentially expressed genes in the TCGA-LGG, GSE80336 and GSE78936 datasets, the second condition was relaxed (base Mean > 50) due to the presence of more genes with very low read count.

Biological processes were analyzed using the PANTHER functional classification system (http://www.pantherdb.org) [55].

### Gene expression correlation heatmaps

To determine the co-expression relationships between genes, Pearson correlation between the expression profiles of a pair of genes was calculated using the *pearsonr* function from *scipy.stats* v1.7.3 Python package. The correlation values were computed using log_2_(TPM + 1) normalized gene expression. For each resulting correlation matrix, heatmaps were built using a *Heatmap* function from the *ComplexHeatmap* v2.10.0 R Bioconductor package [76].

### Determination of metagene specific to the Th17 cells and its prognostic efficacy

To evaluate the prognostic efficacy of Th17 cells immune contexture in LGG patients, we considered Th17-signature [40] and assessed the relationship between the expression of the Th17-associated metagene and overall survival of patients. The TCGA-LGG dataset was used to generate a correlation matrix of gene expression levels from the respective signature by estimating the Pearson’s correlation coefficients. The correlation matrix was subjected to hierarchical clustering (Euclidean distance, average linkage). The metagene associated with LGG-specific Th17 cells was chosen as a cluster of highly correlated genes that included the reliable Th17 cells marker (IL17A) [42]. We then defined metagene expression as the average value of the expression of the genes composing a metagene and assessed the association of the metagene with overall survival. We stratified patients on the basis of the 75th percentile of metagene expression into two groups (high or low expression level). The resulting groups were plotted with respect to overall survival to produce respective Kaplan–Meier curves. Statistical comparison of survival by log-rank Mantel–Cox test was performed between groups. In addition, the median survival (in days) was calculated for each group. We calculated the percent change in median survival (%Δ*MS*) between the high and low metagene expression level groups, as previously reported [14], using the formula $\% \Delta MS = \frac{{MS^{High} - MS^{Low}}}{{MS^{High}}} \times 100$, where *MS*^*High*^ is the median survival in the group with high expression level and *MS*^*Low*^ is the median survival in the group with low expression level.

### Prognostic model construction

A special feature of survival data is right censoring when the observation period expires before death occurs. In this case, the Cox proportional hazards regression model [77] is the most common approach for studying the dependency of a patient’s survival time on several predictor variables.

To identify prognostic genes that affect the survival of patients, univariate Cox proportional hazards regression analysis was performed using *CoxPHFitter* function from *lifelines* v0.26.0 Python package [78]. This method enables the evaluation of the correlation between the expression level of each gene and overall survival in the cohort. The Wald statistic is used to estimate the statistical significance for each of the covariates in relation to overall survival. Only those genes with a *p*-value < 0.05 were considered as significant predictors and entered into the final multivariate Cox regression model. For evaluating the performance of the prediction model, the index of concordance (C-index) was calculated, which is a generalization of the receiver operating characteristic area under curve to survival data that include censored data. The C-index values range from 0 to 1, and the larger value, the better the prediction [79].

Next, we calculated the individual risk score of each patient with coefficient-weighted gene expression and constructed a predictive model with the following formula:

$$Risk\,Score = \mathop {\sum }\limits_{i = 1}^k \left( {Coef_i \times EV_i} \right),$$

where *k* is the number of prognostic genes, *Coef*_*i*_ is the coefficient of the i-th gene in the multivariate Cox regression model, and *EV*_*i*_ is the log_2_(TPM + 1) normalized expression value of the i-th gene. If *Coef*_*i*_ > 0 the i-th gene is defined as a high-risk signature, and if it is < 0, the gene is defined as protective. The patients were divided into high-risk and low-risk groups according to the median risk score calculated based on the prognostic gene signature.

To compare the differences in overall survival time between the low-risk and high-risk patient groups, survival curves were constructed using the Kaplan-Meier method, and the log-rank test was employed to assess the statistical significance of the difference. For this, *KaplanMeierFitter* and *logrank_test* functions from *lifelines* Python package were used. The sensitivity and specificity of the prognostic model for predicting the clinical outcome were evaluated by calculating the area under curve of the time-dependent receiver operating characteristic curve using *survivalROC* function from *survivalROC* v1.0.3 R package [80]. Receiver operating characteristic curves are widely used for presenting the sensitivity and specificity of continuous diagnostic markers for a binary disease outcome. This approach estimates how well the risk score can distinguish those who had an event (died) by a pre-specified time (e.g., 1, 3, 5 years) from those who remained alive.

### Immune-deconvolution: estimation of glioma-infiltrating cells

Immune cell proportions in tissue were estimated using EPIC (Estimating the Proportions of Immune and Cancer cells) deconvolution method [81]. The EPIC method estimates the fraction of five types of immune cells (B cells, CD4^+^ T cells, CD8^+^ T cells, macrophages and NK cells), cancer-associated fibroblasts (CAFs), endothelial cells and uncharacterized cells (mostly cancer cells) from bulk gene expression data. The method is based on the expression profiles of the reference genes for the cell types under consideration and predicts the proportion of these cells and the remaining uncharacterized cells for which no reference profile is given, using constrained least squares regression. Although EPIC is more limited in the number of tested immune cell types compared to other methods [82, 83], it allows for the quantification of non-immune cell types such as CAFs and endothelial cells and was also specially designed for RNA-seq data, not microarray data.

The TPM normalized expression data of the TCGA-LGG dataset were used for estimation of the fractions of cell types in the tumor for each individual, which were deconvoluted by the *EPIC* v1.1.5 R package (https://github.com/GfellerLab/EPIC) [81]. The correlation between risk score of prognostic signature and cell infiltration was calculated using Pearson’s correlation.

### Statistical Analysis

Statistics were calculated in GraphPad Prism (V.9.2). The samples or mice have never been excluded from the analysis. The method of randomisation has not been used in the manuscript. The results of the phagocytosis assay and DC activation and maturation assay were analyzed by two-way ANOVA with Tukey’s multiple comparisons test. Kaplan–Meier survival curves show the timeline of tumor development. Survival in the low-risk and high-risk groups was analyzed by log-rank Mantel–Cox test. The similarity of the variance between the samples in the large groups has been pre-checked with the Levene test.

## Supplementary information

Legend for the suppl. figures

Suppl.Figure 1

Suppl.Figure 2

Suppl.Figure 3

Suppl.Figure 4

checklist

## Data Availability

The RNA-seq data of the DCs co-cultured with glioma GL261 cells generated during the current study are available in the NCBI Gene Expression Omnibus (GEO) repository under accession number GSE218081.

## Code availability

Computer code used in the analyses is available on GitHub (https://github.com/VershininaOlga/Analysis-of-glioma-RNA-Seq-data).
