# Supplementary material for: DC vaccines loaded with glioma cells killed by photodynamic therapy induce Th17 anti-tumor immunity and provide a four-gene signature for glioma prognosis
Source: Cell Death Dis. 2022 Dec 21;13(12):1062. doi: 10.1038/s41419-022-05514-0 (PMC9767932; doi:10.1038/s41419-022-05514-0)
Supplement: Supplementary file 1 — Legend for the suppl. figures [file 41419_2022_5514_MOESM1_ESM.docx]

**Supplementary figures**

**Suppl. Figure 1.** PS-PDT induces mixed-type cell death (**A-B)**. Analysis of cell death by MTT assay in GL261 cells subjected to PS-PDT **(A)** or 2.0 µM mitoxantrone (MTX) **(B)** in the presence/absence of 25 μM zVAD-fmk, 20 μM Nec-1s, 1 μM Fer-1, or 10 μM DFO. **(C)** Prophylactic vaccination of mice was performed by injecting them on day 0 with GL261 cells in the left flank. The GL261 cells were previously treated with 2.0 µM mitoxantrone (MTX) or subjected to three freeze and thaw cycles (F/T) or treated with 1.4 µM photosens (PS) for 4 h and then exposed to a light dose of 20 J/cm^2^ (PS-PDT). Injection of mice with PBS was used as a control. Seven days later, the mice were challenged by subcutaneous injection of viable GL261 cells in the right flank. **(D)** The appearance of the tumors (% survival) at the challenge site and the tumor size **(E)** in mice vaccinated and challenged with GL261 cells as described in **(C).** *Statistically significant difference from the PBS group, p < 0.05; ^#^statistically significant difference from the F/T group, p < 0.05, Wilcoxon test.

**Suppl. Figure 2. (A)** Experimental setup for the prophylactic vaccination of mice injected once on day 0 with GL261 cells treated with three freeze/thaw cycles (F/T), 2.0 µM MTX, or PS-PDT, or with PBS as control, and challenged by intracranial stereotactic injection with viable GL261 cells seven days later. **(B)** Survival of mice vaccinated and challenged. N = 5–6 per group. Statistical significance was determined by logarithmic Mantel-Cox test, *p < 0.05. **(C)** Analysis of the phagocytosis of dying GL261 cells treated with PS-PDT or 2.0 µM MTX and of viable GL261 cells by bone marrow-derived dendritic cells (DCs) in ratios of 1:2 or 1:5 for 2 h. Representative dot plots of phagocytosis assay using CD11c^+^ DCs and 5-chloromethylfluorescein diacetate (CMFDA) tracer-labeled GL261 cells. The experiment was repeated six times. Statistical significance was determined by unpaired Mann-Whitney U test, *p < 0.05.

**Suppl. Figure 3. DC vaccines are protective in a therapeutic setup in the orthotopic glioma mouse model.** (**A**) Experimental setup for the therapeutic vaccination of mice intracranially injected on day 0 with 2 x 10^4^ viable GL261 glioma cells. Thereafter, they received intraperitoneal injections of a suspension containing 1 × 10^6^ prepared DCs on days 2, 6, 10 and 17. DC-based vaccines were prepared by loading the DCs with GL261 cells treated with PS-PDT (PS at a dose of 1.4 µM), 2.0 µM mitoxantrone (MTX) or mice were injected with PBS as described above. (**B**) Survival of mice as described in **(A)**. *p < 0.02, Log-rank (Mantel-Cox) test. (**C**) The neurological status of the mice was assessed every 2–4 days for up to day 60. Shown are the percentages of mice exhibiting neurological alterations (grades 0-4; n = 8-9 per group). (**D**) The temporal progression of neurological deficits in mice treated as described in **(A)** is shown for each group (n = 5-9). *p < 0.05, a statistically significant difference from the PBS group; Multiple t test. (**E**) *Ex vivo* representative T2-weighted MRI images of layer-by-layer frontal brain sections on day 37. The tumor mass is indicated by red arrows. (**F**) Histological analysis of brain sections on day 37. (**G**) Immune cell phenotyping of the isolated draining lymph nodes of therapeutically vaccinated mice. The lymph nodes of mice vaccinated with DC-GL261_PS-PDT contained a significantly increased number of CD8^+^ T cells compared to the control while the number of DCs and macrophages remained unchained (on day 37); n = 3-5 mice; p < 0.04, Kruskal-Wallis test.

**Suppl. Figure 4.** (**A**) Biological processes induced in DCs cocultured with dying glioma GL261 cells pulsed with GL261_PS-PDT. Differentially expressed genes (1242 upregulated, 115 downregulated) categorized by the processes are shown. The circle size is proportional to the number of differentially expressed genes. **(B, C)** The Th17-associated metagene and its relationship with overall survival of TCGA-LGG patients. **(B)** The metagene associated with Th17 cells in the TCGA-LGG dataset (highlighted with yellow lines). Pearson’s correlation coefficient used to analyze the gene co-expression is indicated in the color-coded legend. Red indicates a very strong positive correlation, black no correlation and green a very strong negative correlation. **(C)** The Kaplan-Meier plot of a patient's overall survival (OS, Y-axis) *versus* number of days to death or censoring (X-axis). The TCGA-LGG patients were divided by the 75^th^ percentile of metagene expression into high and low expression groups. Shown are the respective log-rank Mantel-Cox test p-value, median survival in the high (MS^High^) and low (MS^Low^) expression groups, and difference in percent change in median survival (%∆MS) between groups. Two black dotted lines represent the approximate point of two- year and five-year survival (y.s.).
