# Supplementary figures and images for: DC vaccines loaded with glioma cells killed by photodynamic therapy induce Th17 anti-tumor immunity and provide a four-gene signature for glioma prognosis
Source: Cell Death Dis. 2022 Dec 21;13(12):1062. doi: 10.1038/s41419-022-05514-0 (PMC9767932; doi:10.1038/s41419-022-05514-0)

## Slide 1
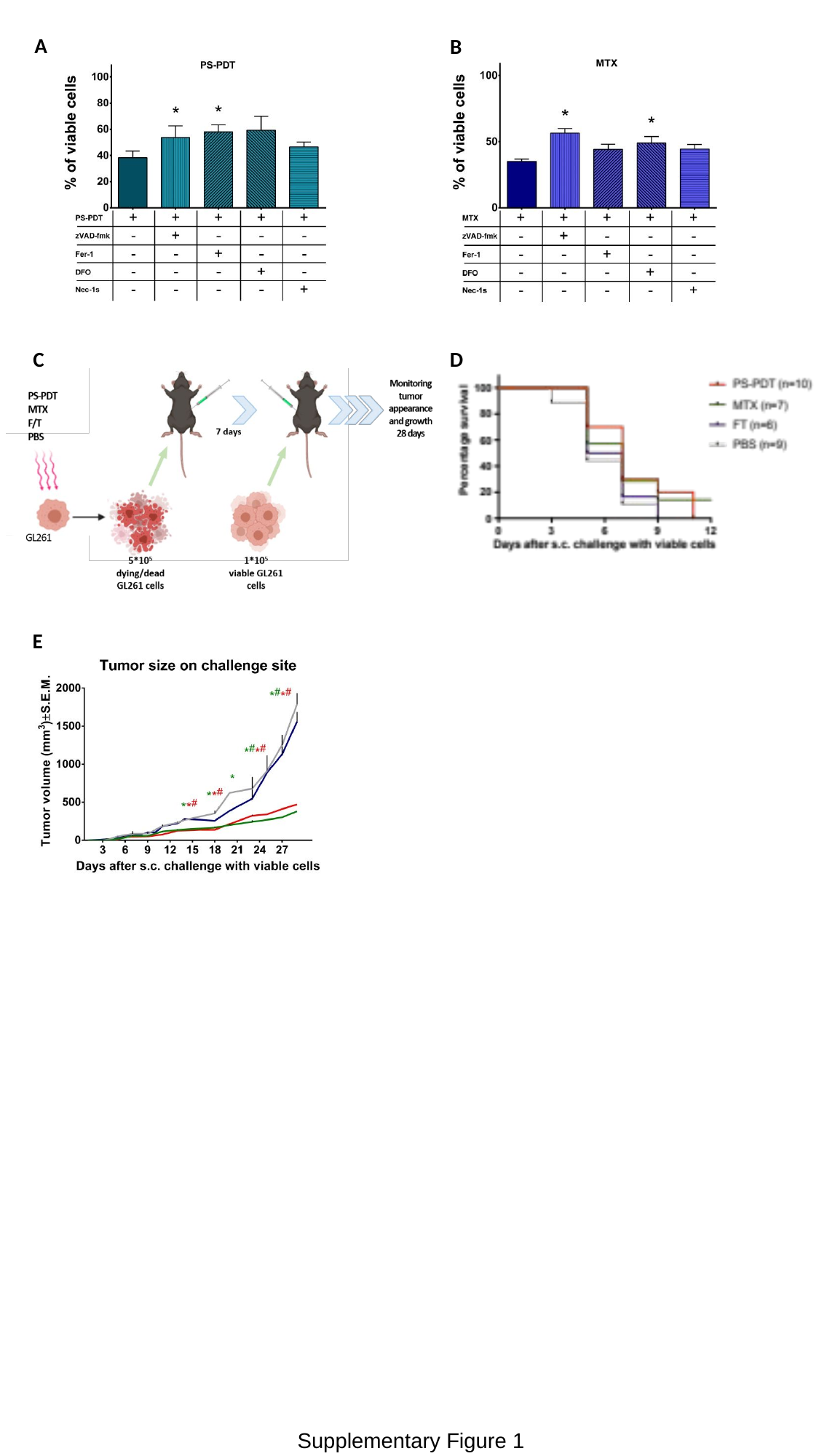

A
B
C
D
E
Supplementary Figure 1

Supplement: Supplementary file 2 — Suppl.Figure 1 [file 41419_2022_5514_MOESM2_ESM.pptx]

## Slide 1
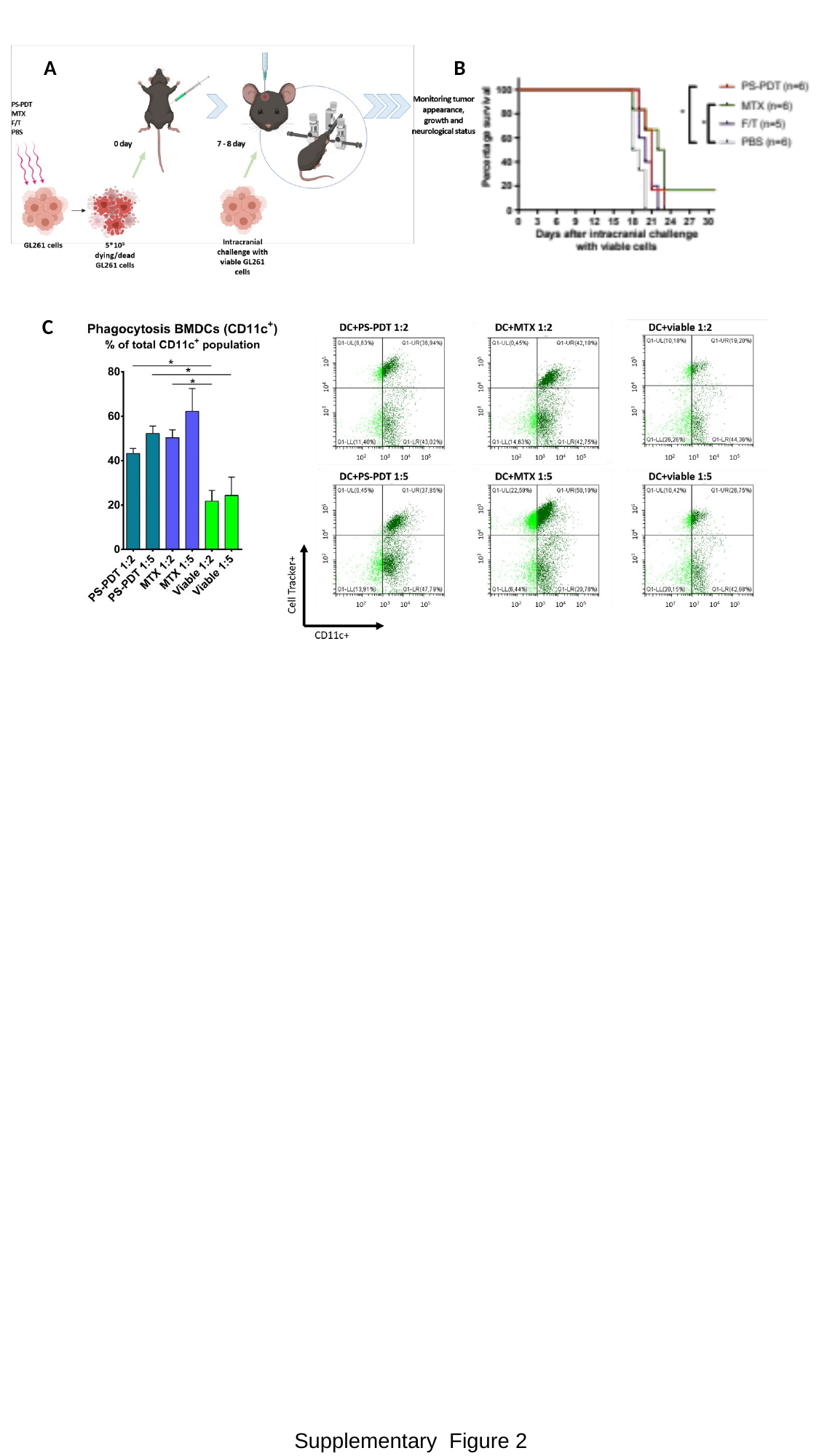

A
B
C
Supplementary Figure 2

Supplement: Supplementary file 3 — Suppl.Figure 2 [file 41419_2022_5514_MOESM3_ESM.pptx]

## Slide 1
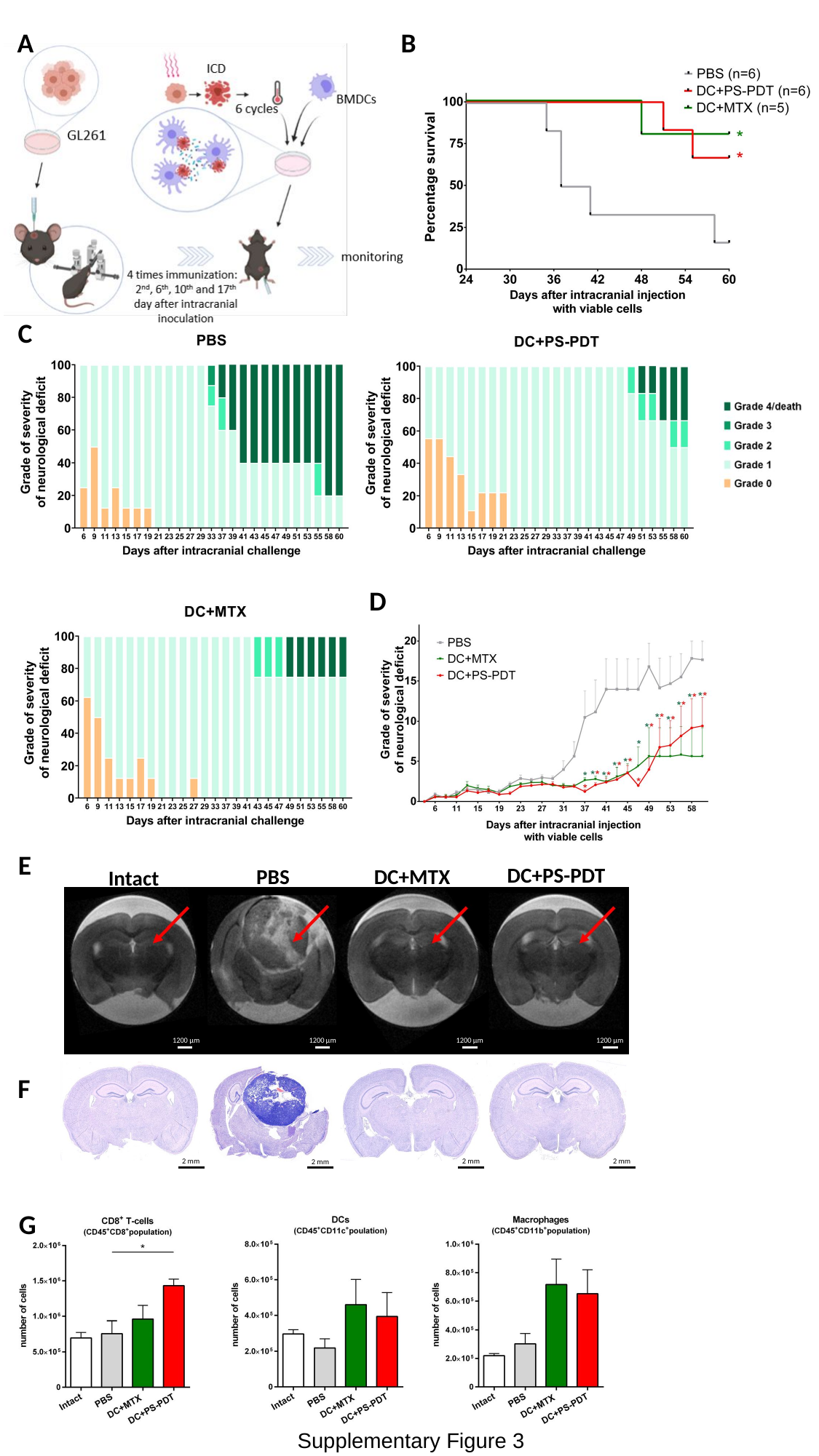

А
B
C
D
E
DC+PS-PDT
PBS
DC+MTX
Intact
1200 µm
1200 µm
1200 µm
1200 µm
F
2 mm
2 mm
2 mm
2 mm
G
Supplementary Figure 3

Supplement: Supplementary file 4 — Suppl.Figure 3 [file 41419_2022_5514_MOESM4_ESM.pptx]

## Slide 1
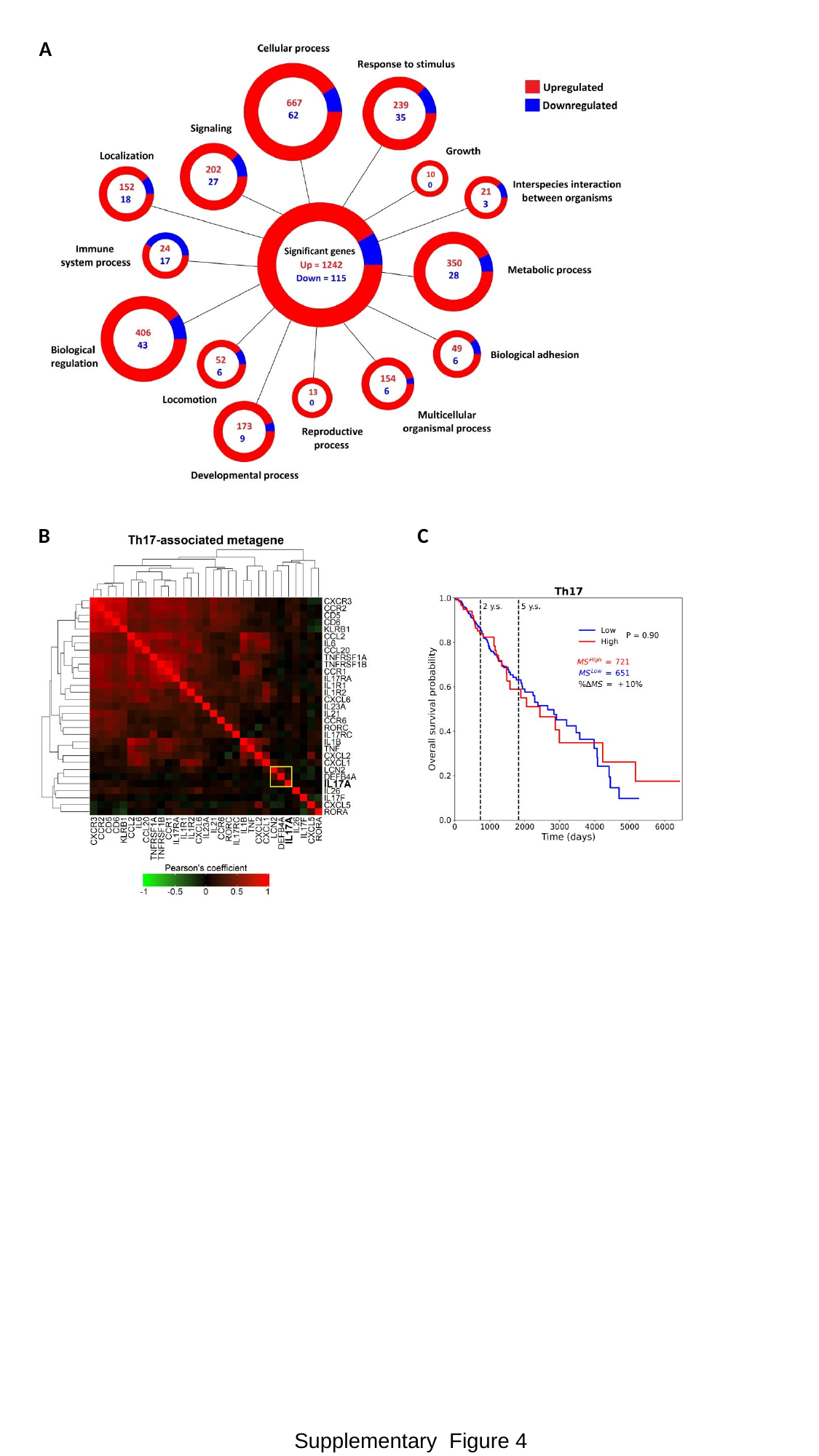

A
B
C
Supplementary Figure 4

Supplement: Supplementary file 5 — Suppl.Figure 4 [file 41419_2022_5514_MOESM5_ESM.pptx]
